# Spatial synaptic connectivity underlies oligodendroglioma evolution and recurrence

**DOI:** 10.21203/rs.3.rs-6299872/v1

**Published:** 2025-04-04

**Authors:** David Raleigh, Kanish Mirchia, Sena Oten, Thiebaud Picart, Minh Nguyen, Vardhaan Ambati, Harish Vasudevan, Jacob Young, Jennie Taylor, Saritha Krishna, David Brang, Joanna Phillips, Arie Perry, Mitchel Berger, Susan Chang, John de Groot, Shawn Hervey-Jumper

## Abstract

Oligodendrogliomas are initially slow-growing brain tumors that are prone to malignant transformation despite surgery and cytotoxic therapy. Understanding of oligodendroglioma evolution and new treatments for patients have been encumbered by a paucity of patient-matched newly diagnosed and recurrent tumor samples for multiplatform analyses, and by a lack of preclinical models for interrogation of therapeutic vulnerabilities that drive oligodendroglioma growth. Here we integrate spatial and functional analyses of tumor samples and patient-derived organoid co-cultures to show that synaptic connectivity is a hallmark of oligodendroglioma evolution and recurrence. We find that patient-matched recurrent oligodendrogliomas are enriched in synaptic gene expression programs irrespective of previous therapy or histologic grade. Analyses of spatial, single-cell, and clinical data reveal epigenetic misactivation of synaptic genes that are concentrated in regions of cortical infiltration and can be used to predict eventual oligodendroglioma recurrence. To translate these findings to patients, we show that local field potentials from tumor-infiltrated cortex at the time of resection and neuronal hyperexcitability and synchrony in patient-derived organoid co-cultures are associated with oligodendroglioma proliferation and recurrence. In preclinical models, we find that neurophysiologic drugs block oligodendroglioma growth and pathologic electrophysiology. These results elucidate mechanisms underlying oligodendroglioma evolution from an indolent tumor to a fatal disease and shed light on new biomarkers and new treatments for patients.

## Introduction

Diffuse infiltrating gliomas with mutations in isocitrate dehydrogenase (IDH) comprise approximately 25% of primary intraparenchymal brain tumors in adults[1]. IDH-mutant gliomas are subdivided into oligodendroglioma with chromosome 1p/19q-codeletion and astrocytoma with *ATRX* and *TP53* mutations[2–4]. Oligodendrogliomas can be associated with particularly long survival after treatment with surgery, ionizing radiation, and chemotherapy[5–7], making identification of serial patient-matched samples for multiplatform molecular analyses challenging. Nevertheless, most IDH-mutant gliomas eventually undergo malignant transformation and recur despite supramaximal resection and cytotoxic therapy[8,9]. Small molecule inhibitors of mutant IDH induce lineage differentiation and may revolutionize the treatment of IDH-mutant gliomas[10,11], but the impact on long-term survival and the efficacy of this treatment in tumors that have undergone malignant transformation may be limited [12] and at least 50% of patients who are treated with mutant IDH inhibitors develop recurrent disease[10]. Thus, there is an unmet need for improved understanding of IDH-mutant glioma evolution so that new biomarkers and new treatments can be developed.

IDH-mutant glioma recurrence and malignant transformation are associated with increased tumor proliferation, *CDKN2A* homozygous deletion, tumor cell de-differentiation, temozolomide (TMZ)-induced hypermutation, and epigenetic reprogramming[13–21]. The extent to which these findings can be targeted and are specific to oligodendroglioma or are more broadly relevant to IDH-mutant glioma is incompletely understood. IDH-wildtype glioblastoma remodeling of human neural circuits decreases survival[22–27], and IDH-mutant gliomas contain tumor cells that demonstrate properties of neurons and glia and fire single, short action potentials[28], but it is unknown if synaptic connectivity contributes to malignant transformation of IDH-mutant gliomas. To address these limitations in our understanding of IDH-mutant glioma biology, we performed histologic, genomic, spatial, electrophysiologic, functional, and pharmacologic analyses of patient-matched newly diagnosed and recurrent oligodendroglioma and astrocytoma samples and patient-derived organoid co-culture models. Our results reveal synaptic connectivity is a hallmark and therapeutic vulnerability that underlies oligodendroglioma evolution and recurrence, shedding light on new biomarkers and new treatments for patients.

## Experimental design and workflow

To identify genomic and cellular mechanisms underlying oligodendroglioma recurrence and evolution, a discovery cohort of 33 patient-matched newly diagnosed and recurrent oligodendroglioma samples from 16 patients were retrospectively identified from the UCSF Brain Tumor Center Biorepository and Pathology Core (Fig. 1a, b, Supplementary Table 1). Median progression free survival (PFS) between serial resections in the oligodendroglioma discovery cohort was 2.4 years (range 0.7 to 7.6 years). Core selection for analysis of all samples was guided by the most representative areas of tumor grading and morphological heterogeneity on whole mount formalin-fixed, paraffin-embedded (FFPE) sections. Histologic examination using hematoxylin and eosin (H&E) staining, immunohistochemical (IHC) staining, and molecular assessment using a targeted next generation DNA sequencing (NGS) panel were performed on all samples in compliance with the 5th edition (2021) of the World Health Organization (WHO) Classification of Tumors of the Central Nervous System (CNS)[2]. All samples were analyzed using IHC for IDH1 R132H mutant protein, ATRX, and Ki67 to measure cell proliferation. Targeted NGS for 529 genes, included assessment of single nucleotide and structural variants in *IDH1, IDH2*, *TERT* including the promoter region, *ATRX*, *CIC*, *FUBP1*, *NOTCH1-3*, *TP53*, and genome wide copy number alterations (CNAs)[29] was performed on all samples. The oligodendroglioma samples in the discovery cohort were additionally analyzed using spatial RNA sequencing of 6mm cores across continuous tiled arrays containing 50μm regions with probes targeting the entire protein coding transcriptome[29,30]. Thirty-one oligodendroglioma samples meeting quality control criteria for spatial RNA, library preparation, and sequencing were retained for downstream analyses.

The oligodendroglioma discovery cohort was comprised of 15 CNS WHO grade 2 and 16 grade 3 oligodendroglioma samples, which were stratified for patient-matched temporal progression across histologic grades (n = 5 grade 2 to 2, n = 5 grade 2 to 3, n = 6 grade 3 to 3) and treatments between serial resections (n = 10 surgical monotherapy, n = 2 TMZ/RT, n = 2 TMZ, n = 1 IDH inhibition, n = 1 other) (Fig. 1a, b, Supplementary Table 1). Spatial transcriptomes were aligned to H&E images using unique oligonucleotide barcodes corresponding to array positions, and the Harmony bioinformatic pipeline was used for sample integration and batch-correction[31], as batch effects can limit analysis of spatial RNA sequencing[29,30]. Uniform manifold approximation and projection (UMAP) analysis of 55,737 spatial transcriptomes demonstrated 15 spatial gene expression programs across the 31 oligodendroglioma samples in the discovery cohort (Supplementary Table 2). Seven spatial gene expression programs were enriched in marker genes associated with diffuse glioma tumor cell states (C0, C1, C2, C5, C6, C7, C8), 4 spatial gene expression programs were enriched for marker genes of non-tumor microenvironment cell types (C3, C4, C7, C12), and 6 spatial gene expression programs were mostly restricted to a single sample in the oligodendroglioma discovery cohort and comprised a minority of spatial transcriptomes (C8, C9, C10, C11, C13, C14) (Extended Data Fig. 1a-c, Extended Data Fig. 2a, Supplementary Table 3). Spatial CNAs were defined using the inferCNV and spatialinferCNV bioinformatic pipelines[32,33], and the output heatmap matrix was binarized to delineate spatial barcodes with chromosome 1p/19q codeletion (Supplementary Table 4). Following initial unsupervised clustering, each spatial transcriptome was manually annotated as tumor (oligodendroglioma CNS WHO grade 2 or 3) or tumor microenvironment using H&E images, IDH1 R132H IHC images, and CNA results (Fig. 1c, Supplementary Table 4). Tumor microenvironment annotations were further separated into spatial gene expression programs that were identified during unsupervised clustering to provide granular spatial transcriptome definitions that were inclusive of histologic characteristics, tumor grade, and gene expression programs (Fig. 1d, e, Supplementary Table 4).

The specificity of findings from the oligodendroglioma discovery cohort were validated using immunofluorescence (IF) microscopy in a separate cohort of 5 newly diagnosed oligodendroglioma samples without documented recurrence after surgical monotherapy and long magnetic resonance imaging follow-up (range 6 to 11 years) (Fig. 1a, b). Specificity was also validated using spatial RNA sequencing and IF microscopy in 16 patient-matched newly diagnosed and recurrent astrocytoma samples from 8 patients (Fig. 1a, b). Astrocytomas in the specificity cohort were comprised of 4 CNS WHO grade 2, 2 grade 3, and 2 grade 4 cases, which were stratified for patient-matched temporal progression across histologic grades (n = 1 grade 2 to 2, n = 1 grade 2 to 3, n = 1 grade 2 to 4, and n = 1 grade 3 to 4). All oligodendroglioma and astrocytoma cases in the specificity cohort were analyzed using the same histologic, IHC, and targeted NGS assays as the discovery cohort (Supplementary Table 1).

Oligodendroglioma single-cell assay for transposase-accessible chromatin (ATAC) sequencing data[17], and bulk RNA sequencing and clinical data from The Cancer Genome Atlas (TCGA)[34], were used to elucidate epigenetic mechanisms and the generalizability of genomic and cellular mechanisms underlying oligodendroglioma recurrence and evolution, respectively. The functional implications of these findings were validated in patients with newly diagnosed or recurrent oligodendroglioma using intraoperative subdural electrocorticography of tumor-infiltrated cortex, and a novel patient-derived organoid co-culture model of IDH-mutant glioma interactions with cortical neurons that was used for electrophysiology, calcium imaging, cell proliferation, IF, and pharmacologic experiments.

## Oligodendroglioma spatial transcriptomes evolve from NPC-like and proneural NPC-like cell states

Oligodendroglioma single-cell RNA sequencing studies support a cancer stem cell model that is similar to IDH-wildtype glioblastoma[11,17,18,21,35,36]. These studies show that oligodendrogliomas are primarily comprised of astrocyte-like (AC-like) and oligodendrocyte-like (OC-like) cell states plus a small number of undifferentiated cells with gene expression programs that are similar to mouse neural stem cells and human neural progenitor cells (NPCs), and are hypothesized to represent a stem-like state which fuels oligodendroglioma growth and recurrence[11,21]. The distribution of oligodendroglioma cell states across spatial transcriptomes during tumor evolution and the proximity of these cell states to microenvironment cell types is unknown.

To address these limitations, oligodendroglioma cell states[21] were deconvolved from spatial transcriptomes in the oligodendroglioma discovery cohort (Fig. 2a-c, Extended Data Fig. 2a, Supplementary Table 3), demonstrating AC-like and OC-like tumor cell states (C1), and a small population of proneural NPC-like cells (C5) that comprised 5.2% of the total number of spatial transcriptomes (Supplementary Table 2). Histologic examination suggested that several tumor-specific spatial gene expression programs in patient-matched newly diagnosed and recurrent oligodendrogliomas could not be deconvolved into published oligodendroglioma cell states (Fig. 2c), but deconvolution of spatial transcriptomes using IDH-wildtype glioblastoma single-cell states[35] revealed AC-like (C1, C6, and a subset of C2), oligodendrocyte progenitor cell-like (OPC-like, C0, C1, and a subset of C2), mesenchymal-like (MES-like, C8), and NPC-like states (C0, C5, and a subset of C2) (Fig. 2d). Cell signature gene set concordance using the 100 most differentially expressed genes per spatial transcriptomic cluster validated enrichment of OPC-like genes in C0 (*SOX6, SOX8, LHFPL3, PDGFRA, PTPRZ1, ANGPTL2*), proneural genes in C0 and C5 (*BCAN, OLIG2, GPR17, SEZ6L, SCG3, CASK, C1QL1*), and NPC-like genes in C2 and C5 (*SOX4, MAP2, DLL1, SOX11, CD24, SNAP25, KIF5A, ATP1B1, STMN2*) (Fig. 2b, Supplementary Table 3). Cycling cells were evenly distributed across spatial transcriptomes (Supplementary Table 4), and histologic examination of spatial transcriptomes demonstrated mature neurons and rare oligodendroglioma tumor cells in C2, mature neurons and tumor cells in C5, tumor cells in C0, C6, C10, and C11, low cellularity tumor cells in C1, tumor cells with a mesenchymal phenotype in C8 and C9, intravascular and extravasated red blood cells in C3, thin and thick-walled blood vessels in C4, tumor cells and cortical neurons at the invasive/infiltrative edge of oligodendrogliomas in C7, and an admixture of normal grey or white matter and scattered infiltrating tumor cells in C12, C13, and C14 (Fig. 2e). Trajectory analyses using unsupervised monocle-based pseudotime and RNA velocity with latent and dynamic models[37–39] suggested that NPC-like (C2) and proneural NPC-like (C5) spatial transcriptomes represented the initial steps in oligodendroglioma evolution across patient-matched newly diagnosed and recurrent tumor samples (Fig. 2f, g). In support of this hypothesis, there was a mixture of 1p/19q-intact and 1p/19q-codeleted spatial transcriptomes in the NPC-like cluster (C2) (Fig. 1d).

### Spatial synaptic gene expression programs underlie oligodendroglioma evolution

The 2021 WHO classification of CNS tumors retains a two-grade system for oligodendroglioma, and like prior WHO grading schemes, the distinction between oligodendroglioma grades relies on histologic criteria such as mitotic activity[2,40]. Oligodendroglioma treatment paradigms are equivalent across genomic features[6,7,41], including tumors with *PIK3CA* mutations or *CDKN2A* homozygous deletion that are associated with worse clinical outcomes in some cases[3,42–45], tumors with diverse CNAs or elevated mutation burden that do not provide prognostic or predictive information[46], and tumors from the rare oligosarcoma epigenetic group that is associated with worse clinical outcomes[47]. Thus, definitive molecular criteria for therapeutic response stratification or for distinguishing CNS WHO grade 2 and grade 3 oligodendroglioma do not exist.

To determine if subclonal spatial CNAs were associated with cell state evolution in newly diagnosed versus recurrent oligodendroglioma samples, inferCNV was used to define spatial copy number gains or losses in the oligodendroglioma discovery cohort (Fig. 3a). Unsupervised hierarchical clustering revealed 7 distinct spatial CNA clones (Fig. 3a, b), 2 of which only contained chromosome 1p/19q-codeletion without additional CNAs (CNA1, CNA2). The smallest CNA clone, which accounted for 5.0% of spatial transcriptomes (CNA4), showed marked aneuploidy with copy number gains and losses across most chromosomes (Fig. 3a, b). There were no significant associations between spatial CNA clones and CNS WHO grade transition or newly diagnosed versus recurrent oligodendroglioma presentation, but when stratifying oligodendrogliomas by treatments between serial resections, recurrent tumors after treatment with TMZ with or without radiotherapy (RT) were comprised of spatial CNA clones with lower total CNA burden (CNA0, CNA1, CNA2, CNA3) (Fig. 3c). In contrast, oligodendrogliomas that were treated with surgical monotherapy contained CNA clones with high total CNA burden at the time of recurrence (CNA4, CNA5, CNA6).

Irrespective of spatial cell states (Fig. 2a-g), spatial CNA clones (Fig. 3a-c), treatments between serial resections (Fig. 3d-f), or CNS WHO grade transitions between patient-matched newly diagnosed and recurrent samples (Fig. 4a-d), oligodendroglioma spatial transcriptomes showed convergent enrichment in synaptic gene expression programs at the time of recurrence. Differential gene expression and ontology analyses demonstrated modulation of ionic transport, synapse organization, and synaptic plasticity including modulation of chemical synaptic transmission (*LRRN1, SLC17A7, PTPRT, DAG1, PDLIM5*), regulation of trans-synaptic signaling (*SLC12A4, ALDH5A1, NR4A1, THBS1, GLUD1*), and regulation of neuron projection development (*NEFL, PHGDH, SEMA5A, NEFL, ASTN2*) in recurrent versus newly diagnosed oligodendroglioma samples after surgical monotherapy, TMZ, or RT/TMZ (Fig. 3d-f). There were no significant differences in oligodendroglioma cell states in recurrent versus newly diagnosed samples according to treatments between serial resections (Supplementary Table 4), and cell states during CNS WHO grade transitions were heterogeneous. Recurrent CNS WHO grade 3 oligodendrogliomas showed an increase in NPC-like (C2) and proneural NPC-like (C5) spatial transcriptomes (Supplementary Table 4), a finding that is consistent with expanded NPC-like cells in association with malignant transformation in oligodendroglioma[17,21]. There was also a decrease in NPC-like cell states in tumors that presented as CNS WHO grade 2 at initial diagnosis and recurred as grade 2, and a decrease in proneural OPC-like cell states in tumors that presented as CNS WHO grade 3 at initial diagnosis and recurred as grade 3 (Fig. 4a, b, Supplementary Table 4). Despite these heterogeneous differences in cell state evolution, differential gene expression and ontology analyses again revealed convergent modulation of chemical synaptic transmission (*DNM1, NEFL, NCDN, CAMK2A*), regulation of trans-synaptic signaling (*SYN1, SNCA, SNCB, SNAP25, MBP, SLC17A7, KIF5A*), and regulation of axon or neuron development (*STMN2, CCK, CHN1, UCHL1, PACSIN1*) in recurrent versus newly diagnosed oligodendroglioma samples irrespective of CNS WHO grade at the time of initial diagnosis or at recurrence (Fig. 4c, d).

Knowledge of biological pathways underlying diverse cancers has generated robust targeted gene expression biomarkers that are recommended for risk stratification and prediction of treatment response by the National Comprehensive Cancer Network (NCCN)[41,48–52]. To shed light on biomarkers of oligodendroglioma recurrence, the 100 most differentially expressed genes contributing to the convergent synaptic ontologies underlying oligodendroglioma evolution and recurrence were investigated for overlap in oligodendrogliomas that (1) presented as CNS WHO grade 2 at initial diagnosis and recurrence, (2) transformed from grade 2 to grade 3 at recurrence, or (3) presented as grade 3 at initial diagnosis and recurrence, irrespective of treatment with (4) surgical monotherapy, (5) RT/TMZ, or (6) TMZ between serial resections. From the list of overlapping genes, a 28-gene connectivity score was compiled using knowledge of biological pathways and cellular processes underlying CNS homeostasis and glioma evolution (Supplementary Table 5). Among the genes comprising the connectivity score, *THBS1* encodes TSP1, an extracellular matrix (ECM) molecule that is regulated by TGFβ, is secreted by immature and developing astrocytes to facilitate synapse formation, has a direct role in ECM connectivity at the edges of gliomas, is associated with higher grade gliomas and shorter survival, and may have a role in invasion and angiogenesis during glioma progression[22,53–56]. *GJA1* encodes connexin 43 (Cx43), which forms intercellular gap junctions, mediates communications between glioma cells and neurons, and drives synaptic plasticity, synaptic depolarization, and neuronal activation[26,57,58]. *NDRG2* regulates astrocytic glutamate transport and contributes to sodium-dependent excitatory amino acid transporter (EAAT) uptake on astrocytes to maintain synaptic homeostasis[59,60]. Additional biological context for all 28 genes comprising the connectivity score is provided in Supplementary Table 5.

To reduce variability from low-ranking genes and maintain score consistency across different datasets, the Mann-Whitney U statistic and UCell package[61] were used to calculate aggregate expression of the 28-gene connectivity score in spatial transcriptomes from the oligodendroglioma discovery cohort. Tumor hemorrhage and vascular clusters (C3, C4) had the lowest average connectivity score, and the highest average connectivity score was in seen spatial transcriptomes containing oligodendroglioma cells (C0, C1, C2, C5, C6, C7) (Fig. 5a). Moreover, the connectivity score was enriched in recurrent compared to newly diagnosed oligodendroglioma samples, a finding that was driven by increased averaged expression in AC/OC-like (C1), proneural NPC-like (C5), and infiltrative edge (C7) spatial transcriptomes, with no significant increase in proneural OPC-like or NPC-like cell states (C0, C2) at the time of recurrence (Fig. 5a).

The average connectivity score per spatial transcriptome was integrated with tissue histology and categorized into areas of (1) distant normal cortex, including neocortical brain parenchyma with no evidence of infiltrating tumor cells on H&E or IHC for IDH1 R132H, (2) white matter with infiltrating tumor cells, (3) cortex with infiltrating tumor cells, (4) densely cellular oligodendroglioma CNS WHO grade 2, and (5) densely cellular oligodendroglioma CNS WHO grade 3. Distal normal cortex had the lowest average connectivity score, and there was a progressive increase in average connectivity score from white matter with infiltrating tumor cells to densely cellular oligodendroglioma CNS WHO grade 2 to densely cellular oligodendroglioma CNS WHO grade 3 to cortex with infiltrating tumor cells (Fig. 5b, c). Most recurrent oligodendroglioma spatial transcriptomes showed higher connectivity scores than newly diagnosed oligodendroglioma spatial transcriptomes in patient-matched samples, but in some instances, newly diagnosed spatial transcriptomes showed high connectivity scores that remained elevated at the time of recurrence (Fig. 5b). IF staining for TSP1, NDRG2, and Cx43 revealed a similar distribution, with the lowest expression in distal normal cortex, slightly higher expression in areas of white matter with infiltrating tumor cells, intermediate expression in densely cellular oligodendroglioma CNS WHO grade 2, and the highest expression in regions of densely cellular oligodendroglioma CNS WHO grade 3 and cortex with infiltrating tumor cells (Fig. 5d, e).

There were no recurrent DNA mutations or CNAs on targeted NGS (Supplementary Table 1) or spatial analyses (Fig. 3a-c) that could account for enriched synaptic gene expression in CNS WHO grade 3 versus grade 2 oligodendrogliomas (Fig. 5c, e). Thus, to shed light on epigenetic mechanisms potentially contributing to synaptic gene expression in oligodendroglioma, single-cell ATAC sequencing data from CNS WHO grade 2 (n = 2) and grade 3 (n = 2) oligodendrogliomas were interrogated[17]. Genome wide peak calling showed higher mean transcriptional start site (TSS) enrichment across all protein coding genes in CNS WHO grade 3 versus grade 2 oligodendrogliomas (Extended Data Fig. 3a), a finding that was conserved for the 28 genes comprising the connectivity score (Extended Data Fig. 3b-d). These data suggest that changes in chromatin accessibility during CNS WHO grade transition may contribute to synaptic gene expression in oligodendroglioma.

### Synaptic gene expression programs underlie oligodendroglioma clinical outcomes

To determine if synaptic gene expression was associated with oligodendroglioma clinical outcomes, differential gene expression analysis was performed between spatial transcriptomes from (1) newly diagnosed CNS WHO grade 2 oligodendrogliomas that transformed to grade 3 at the time of recurrence and (2) newly diagnosed CNS WHO grade 2 oligodendrogliomas that did not undergo grade transformation at the time of recurrence (Fig. 6a, Supplementary Table 6). Gene ontology analysis demonstrated modulation of chemical synaptic transmission, regulation of trans-synaptic signaling, and axon development in newly diagnosed oligodendrogliomas from the discovery cohort that underwent

CNS WHO grade transition at the time of recurrence (Fig. 6b). IF staining for TSP1, NDRG2, and Cx43 on a separate cohort of newly diagnosed CNS WHO grade 2 oligodendrogliomas without documented recurrence after surgical monotherapy (n = 5) and long magnetic resonance imaging follow-up (range 6 to 11 years) (Fig. 1a, b) showed low TSP1 and low NDRG2 expression in all regions and bimodal Cx43 expression (Fig. 6c, d). IF staining on newly diagnosed CNS WHO grade 2 oligodendrogliomas from the discovery cohort showed higher Cx43 expression in tumors with eventual recurrence as grade 3 versus grade 2 (Fig. 6d). Among newly diagnosed CNS WHO grade 2 oligodendrogliomas without documented recurrence, 3 cases displayed high Cx43 expression that was comparable to tumors with eventual recurrence as grade 3, and 2 cases displayed low Cx43 expression that was comparable to tumors without eventual CNS WHO grade transition (Fig. 6d). These data suggest that synaptic gene expression could be used as a prognostic biomarker for oligodendroglioma recurrence. To test this hypothesis, IDH-mutant glioma RNA sequencing and clinical data from TCGA and the 28-gene connectivity score (Fig. 5a-c, Supplementary Table 5) were used to develop LASSO and Elastic Net regularized Cox regression models using PFS or overall survival (OS) as endpoints in a training cohort comprised of 326 patients with 10-fold cross validation (Extended Data Fig. 4a). The resulting linearly rescaled continuous synaptic gene expression biomarkers were prognostic for PFS and OS in newly diagnosed oligodendroglioma (Fig. 6e) and astrocytoma (Extended Data Fig. 5b) from an independent validation cohort comprised of 109 patients, and the maximally selected rank statistic identified discrete low versus high connectivity scores that were also prognostic for clinical outcomes in the independent validation cohort (Fig. 6f).

To determine if synaptic gene expression in IDH-mutant glioma was specific to oligodendroglioma or more broadly relevant to astrocytoma, spatial RNA sequencing and IF microscopy was performed in 16 patient-matched newly diagnosed and recurrent astrocytoma samples from 8 patients (Fig. 1a, b, Extended Data Fig. 5a, b). The Harmony bioinformatic pipeline was used for sample integration and batch-correction, and UMAP analysis 10,189 spatial transcriptomes demonstrated 10 spatial gene expression programs across the 16 astrocytoma samples in the specificity cohort (Extended Data Fig. 5c, d, Supplementary Table 7–9). UMAP projection of the 28-gene connectivity score revealed minimal heterogeneity across astrocytoma spatial transcriptomes (Extended Data Fig. 5e). Integration and batch correction of all oligodendroglioma samples from the discovery cohort and astrocytoma samples from the specificity cohort (Extended Data Fig. 6a, b, Supplementary Table 10, 11) demonstrated that CNS WHO grade 2 and grade 3 oligodendroglioma and astrocytoma spatial transcriptomes were overlapping, but that CNS WHO grade 4 astrocytoma spatial transcriptomes were distinct (Extended Data Fig. 6c, d). UMAP projection of the 28-gene connectivity score showed regional heterogeneity with increased expression in CNS WHO grade 3 and 4 spatial transcriptomes compared to grade 2 transcriptomes (Extended Data Fig. 6e). Pseudotime trajectory analysis correlated with the 28-gene connectivity score (Extended Data Fig. 6f), suggesting that synaptic gene expression was associated with IDH-mutant glioma evolution. Nevertheless, IF staining on the astrocytoma specificity cohort showed uniform high expression of TSP1, NDRG2, and Cx43 without enrichment in cortical regions with infiltrating astrocytoma cells (Extended Data Fig. 7a, b). Thus, like oligodendroglioma, astrocytoma spatial transcriptomes are enriched in synaptic gene expression programs (Extended Data

Fig. 5e) that positively correlate with CNS WHO grade transition and tumor evolution (Extended Data Fig. 6b, f) and negatively correlate with clinical outcomes (Fig. 6f, Extended Data Fig. 4b). In contrast to oligodendroglioma, astrocytoma synaptic gene expression is independent of spatial location (Extended Data Fig. 7a, b).

*Recurrent oligodendroglioma remodels human electrophysiology and neurophysiologic drugs block oligodendroglioma growth and pathologic electrophysiology in preclinical models*

IDH-wildtype glioblastoma initiation, proliferation, invasion, and evolution are driven by functional interactions between tumor cells and neurons[22–26,62], but it is unclear if such interactions contribute to IDH-mutant glioma evolution. Spatial transcriptomic (Fig. 3–5), single-cell (Extended Data Fig. 3), and clinical analyses (Fig. 6a-f, Extended Data Fig. 4) suggest that synaptic gene expression programs in regions of cortical infiltration underlie oligodendroglioma proliferation and recurrence. To test the functional implications of these findings in patients, intraoperative subdural electrocorticography measurements of tumor-infiltrated cortex were performed in 8 patients with newly diagnosed CNS WHO grade 2 oligodendroglioma and 2 patients with recurrent grade 2 oligodendroglioma. Local field potentials from 291 electrodes showed that relative connectivity was increased in tumor-infiltrated cortex from recurrent compared to newly diagnosed oligodendroglioma (Fig. 6g, Extended Data Fig. 8a), suggesting that electrical connections between oligodendroglioma cells and neurons may contribute to oligodendroglioma proliferation and recurrence.

To test this hypothesis and interrogate electrophysiological and functional interactions between IDH-mutant glioma cells and neurons, we developed a 3-dimensional fusion model comprised of patient-derived oligodendroglioma (GliO) or astrocytoma (GliA) organoids[63] in co-culture with mouse cortical neurospheres (cNS)[22]. GliO and GliA organoids were derived from 17 patients with CNS WHO grade 2 or grade 3 oligodendroglioma or grade 2, grade 3, or grade 4 astrocytoma to enable comparisons across histologic grades. Multielectrode array (MEA) recordings of cNS with or without fusion to CNS WHO grade 2 or grade 3 GliO organoids were used to assess the effect of oligodendroglioma infiltration on hyperexcitability. Both CNS WHO grade 2 and grade 3 GliO + cNS co-cultures exhibited increased electrophysiologic connectivity, as defined by weighted mean firing rates, synchronization, burst percentages, and burst frequencies compared to cNS alone, and hyperexcitability increased with increasing histologic grade (Fig. 7a, Extended Data Fig. 9a, b). Two-photon calcium imaging was used to validate MEA findings and showed greater spontaneous calcium transients in CNS WHO grade 2 and grade 3 GliO + cNS co-cultures compared to cNS alone (Fig. 7b, Extended Data Fig. 10a, Supplementary Video 1–3). Only CNS WHO grade 3 GliO + cNS co-cultures demonstrated both higher calcium event frequency and reduced inter-spike intervals, suggesting that a gradient of neuronal connectivity underlies oligodendroglioma CNS WHO grade transition. To assess the effect of cNS activity on oligodendroglioma cell proliferation, co-cultures were analyzed using IF for Ki67 and human nuclear antigen (HNA) to distinguish tumor cells from neurons. CNS WHO grade 2 and grade 3 GliO + cNS co-cultures had more double-positive $Ki67^+$ $HNA^+$ cells compared to GliO organoids alone (Fig. 7c), and histologic examination revealed greater oligodendroglioma cell invasion into cNS from grade 3 GliO cocultures than from grade 2 GliO co-cultures (Fig. 7d). IF for Synapsin1 and Homer1 revealed more colocalization of pre- and post-synaptic puncta in CNS WHO grade 2 and grade 3 GliO + cNS co-cultures compared to cNS alone (Fig. 7e). In contrast, GliA + cNS co-cultures did not display any significant changes in neuronal excitability compared to cNS alone (Extended Data Fig. 11a), and only WHO grade 4 GliA + cNS co-cultures demonstrated an increase in double-positive $Ki67^+$ $HNA^+$ cells compared to GliA organoids alone (Extended Data Fig. 11b). These data align with IF from the astrocytoma specificity cohort showing no significant changes in synaptic gene expression in cortical regions with infiltrating astrocytoma cells (Extended Data Fig. 7a, b), and suggest that WHO grade 4 astrocytomas may adopt alternate, non-electrophysiological interactions with cNS that influence tumor cell proliferation.

Among the 28-genes comprising the oligodendroglioma connectivity score (Fig. 5a-c, Supplementary Table 5), TSP1 and Cx43 regulate synaptic plasticity by increased propagation of synaptic depolarization and neuronal inactivation. Pharmacologic targeting of TSP1 and Cx43 was therefore investigated using gabapentin (GBP) for TSP1 inhibition[64], and meclofenamic acid (MFA) for Cx43 inhibition[65–67], which attenuate synapse formation and gap junction signaling, respectively. Treatment of GliO + cNS co-cultures with GBP or MFA reduced MEA measures of hyperexcitability (Fig. 7f, g), although through distinct electrophysiological endpoints. GBP diminished mean firing and bursts (Fig. 7f), and MFA reduced synchrony (Fig. 7g), and both neurophysiologic drugs decreased the number of double-positive $Ki67^+$ $HNA^+$ cells in GliO + cNS co-cultures compared to vehicle treatment (Fig. 7h). Thus, inhibiting electrical connections between oligodendroglioma cells and neurons may be a viable therapeutic strategy to block oligodendroglioma proliferation and recurrence.

## Discussion

There is an unmet need for new biomarkers and new therapies that target genomic and cellular mechanisms driving oligodendroglioma evolution from an indolent tumor to a fatal disease[5–7]. Here we integrate spatial and functional analyses of tumor samples and patient-derived organoid co-cultures to show that synaptic connectivity is a hallmark of oligodendroglioma evolution and recurrence. We overcome longstanding barriers to understanding oligodendroglioma biology by assembling serial patient-matched samples for multiplatform analyses and by developing a preclinical 3-dimensional fusion model to interrogate electrophysiological and functional interactions between tumor cells and neurons. We develop a synaptic gene expression biomarker to predict oligodendroglioma recurrence, test generalizability using TCGA samples, and test specificity in serial patient-matched astrocytoma samples and in newly diagnosed oligodendroglioma samples without documented recurrence after surgical monotherapy. We use single-cell data to show that chromatin accessibility underlies synaptic gene expression in oligodendroglioma and find that neurophysiologic drugs block oligodendroglioma growth and pathologic electrophysiology in preclinical models. These results elucidate mechanisms underlying oligodendroglioma evolution and shed light on new biomarkers and new treatments for patients. In glioblastoma, epigenetic studies reveal neural signatures underlie tumor progression[68,69], and in comparison to neural signatures in glioblastoma, the oligodendroglioma synaptic gene expression biomarker reported here shows overlap in genes involved in synaptic function (*GRIN3A, SYT4, SNAP25*) and neuronal differentiation (*ERC2, SYP, KIF5A*), but no overlap in genes regulating calcium homeostasis. These studies in glioblastoma deconvolve neuronal versus tumor cell populations using a combination of bioinformatic approaches and show maximal association of AC-like cells with high-neural tumors that have the worst clinical outcomes[68,69]. In contrast, we identified maximal oligodendroglioma synaptic connectivity scores in proneural OPC-like cells, suggesting that different signaling mechanisms and cell populations may contribute to phenotypically similar tumor-neuron interactions that contribute to glioblastoma or oligodendroglioma progression and clinical outcomes.

The median PFS between serial resections in the oligodendroglioma discovery cohort in this study was 2.4 years, which suggests that many of the tumors were more aggressive than may be typical for oligodendroglioma. Nevertheless, most tumors in the oligodendroglioma discovery cohort were treated with surgical monotherapy, and it is likely that PFS would have been extended by adjuvant cytotoxic[9] or molecular therapy[10]. The emergence of significant spatial CNA heterogeneity after surgical monotherapy but not adjuvant cytotoxic therapy is unexpected (Fig. 3c) and suggests that divergent clonal evolution may occur in the absence of selective pressure from adjuvant therapy. Understanding how small molecule inhibitors of mutant IDH may influence oligodendroglioma synaptic gene expression will be important as serial patient-matched samples become available. The one sample in this study that developed recurrence after mutant IDH inhibition showed a unique mesenchymal phenotype at the time of recurrence (Fig. 1a, 1b, 2d), but this sample was from a patient with a multiply-recurrent, enhancing oligodendroglioma, and the efficacy of small molecule inhibitors of mutant IDH is limited in tumors that have undergone malignant transformation[12]. Thus, it is difficult to conclude that mesenchymal transition in this sample was associated with mutant IDH inhibition as opposed to innate evolution. Moreover, it is difficult to speculate how synaptic connectivity may relate to oligodendroglioma hypermutation, as no hypermutated tumors were included in either the discovery or the specificity cohort of this study.

The distribution of synaptic gene expression in newly diagnosed oligodendrogliomas without documented recurrence after surgical monotherapy, despite long magnetic resonance imaging follow-up, was bimodal (Fig. 6d). These data suggest that synaptic gene expression (Fig. 6e, f), and perhaps even synaptic connectivity at the time of initial resection (Fig. 6g), can be used to predict not only when oligodendrogliomas will recur, but also whether or not they will undergo CNS WHO grade transition at the time of recurrence. Although some of these findings appear to be conserved in astrocytomas (Extended Data Fig. 5–7) and preclinical GliA + cNS co-culture models (Extended Dat Fig. 11), others are not. Further investigation will be needed to clarify alternate, non-electrophysiological interactions between astrocytoma cells and neurons that may influence tumor cell proliferation and recurrence. In GliO + cNS co-cultures, we show that the neuronal microenvironment drives tumor growth and invasion through a bidirectional process involving neuronal hyperexcitability and tumor cell proliferation (Fig. 7a-e). Repurposed neurophysiologic drugs have proven preclinical benefits in glioblastoma, but the exact mechanism of action for many of these agents remain incompletely understood. Neurotransmitter reuptake or knowledge of primary target genes does not appear to predict drug activity, but there is good correlation between drug efficacy and modulation of downstream signaling pathways[70]. Here we show that pharmacologic disruption of neuronal signaling in oligodendroglioma reduces these tumor-promoting effects (Fig. 7f-h), which underscores the therapeutic potential of targeting tumor-neuron synaptic mechanisms to treat patients with oligodendrogliomas that are resistant to standard interventions, as is the case for glioblastomas. Prospective testing of this therapeutic strategy in patients, and validation of the connectivity score we report in larger cohorts, we will be required to determine if these discoveries are truly practice changing. In the interim, the data reported here builds upon a growing body of literature that shows tumor-neuron interactions are fundamental for the growth and evolution of brain tumors.

## Declarations

### Acknowledgements

We thank Anny Shai and the staff of the UCSF Brain Tumor Center Biorepository and Pathology Core, Mylinh Bernardi and the staff of the Gladstone Institutes Genomics Core, Eric Chow and the staff of the UCSF Center for Advanced Technology, and Mario Suva, Jingyi Wu, and Luis Nicolas Gonzalez Castro. TP was supported by and thanks the Hospices Civils de Lyon (France), the Ligue Nationale Contre le Cancer (France), the Philippe Foundation and the French Society of Neurosurgery (SFNC) for their financial support. This study was supported by grants from NINDS R01 NS137850 and Oligo Nation to SLHJ and the Gianna Rae Meadows Research Fund for Oligodendroglioma to KM, HNV, JSY, JFDG, and DRR.

### Author contributions statement

All authors made substantial contributions to the conception or design of the study; the acquisition, analysis, or interpretation of data; or drafting or revising the manuscript. All authors approved the manuscript. All authors agree to be personally accountable for individual contributions and to ensure that questions related to the accuracy or integrity of any part of the work are appropriately investigated and resolved and the resolution documented in the literature. KM conceived and designed the study with supervision from DRR, analyzed bioinformatic data, and performed and analyzed histologic analyses with supervision from JJP and AP. SO performed co-culture and organoid experiments with supervision from SLHJ, who conceived and designed the co-culture and organoid experiments. TP performed immunofluorescence and microscopy experiments with supervision from KM, SK, SLHJ, and DRR. MPN performed gene expression biomarker informatic analyses with supervision from KM and DRR. VA analyzed human electrophysiology data that were generated and supervised by DB and SLHJ. The study was supervised by KM, HNV, JSY, JWT, MSB, SMC, JFDG, SLHJ, and DRR. The manuscript was prepared by KM, SO, SLHJ, and DRR with input from all authors.

### Competing interests statement

The authors declare no competing interests.

#### Tables

Not applicable.

#### Data availability

DNA sequencing and spatial RNA sequencing data from IDH-mutant glioma samples (n=47) that support the findings in this study have been deposited to the Sequence Read Archive (https://www.ncbi.nlm.nih.gov/sra) under BioProject ID PRJNA1235962. Publicly available oligodendroglioma single-cell ATAC sequencing data were used in this study (GSE241745). The publicly available datasets GRCh37 (hg19, https://www.ncbi.nlm.nih.gov/datasets/genome/GCF_000001405.13/), and GRCh38 (hg38, https://www.ncbi.nlm.nih.gov/datasets/genome/GCF_000001405.40/) were used in this study. Source data are provided with this study. A reviewer link with access to the data prior to release is provided with the submission materials.

#### Code availability

The open-source software, tools, and packages used for data analysis in this study are referenced in the methods where applicable and include 10x Loupe Browser software (v8.1), Aggr (v2.0.0), Seurat R package (v4.3.0), R (v4.2.1), RStudio (v2022.07.2 Build 576), clustree (v0.5.0), Harmony (v0.1.1), inferCNV (v1.14.0), SpatialInferCNV (v0.1.0), monocle3 (v1.3.1), velocyto (v0.17.16), scVelo (v0.2.5), UCell (v2.11.1), SCpubr (v2.0.2), clusterProfiler (v3.2), SCDC (v 0.0.0.9000), Signac (v1.14.0), AutoAnnotate (v1.3.5), Burrows-Wheeler aligner (v0.7.17), Genome Analysis Toolkit (v4.3.0.0), Picard (v2.27.5), Integrative Genome Viewer (v2.16.0), NxClinical (v6.0), Olympus cellSens Standard Imaging (v1.16), Aperio ImageScope (v12.4.3.5008), Nikon NIS-Elements (v5.42.05), FIJI (v2.9.0), and Photoshop (v26.2.0), MATLAB (v2024b), maftools (v2.18.0), survival R package(v3.7-0), survminer(v0.5.0). Custom code was generated and used for analysis of organoid imaging characteristics and is available at https://github.com/mdm247/spatial_diffuseGlioma.git.

## Methods

*Inclusion and ethics*

This study complied with all relevant ethical regulations and was approved by the University of California San Francisco (UCSF) Institutional Review Board (13-12587, 17-22324, 17-23196, 17-23215, and 18-24633). As part of routine clinical practice, all patients included in this study signed a written waiver of informed consent to contribute de-identified tissue for research.

*Study design*

The discovery cohort was comprised of 33 patient-matched newly diagnosed or recurrent oligodendroglioma samples with well-annotated pathologic, molecular, and clinical follow-up data that were retrospectively identified from the UCSF Brain Tumor Center Biorepository and Pathology Core. Oligodendroglioma samples from the discovery cohort were analyzed using spatial RNA sequencing, targeted next-generation DNA sequencing, immunofluorescence (IF) microscopy, histology, and immunohistochemistry (IHC). This cohort was subjected to spatial transcriptomic sequencing and used to identify a set of 28 synaptic connectivity genes that were associated with risk of recurrence and grade transformation. The specificity of findings from the discovery cohort were validated using IF microscopy in a cohort of 5 oligodendrogliomas without documented recurrence but with long interval recurrence-free survival and using spatial RNA sequencing and IF microscopy in 16 patient-matched newly diagnosed or recurrent astrocytoma samples. Publicly available oligodendroglioma single-cell ATAC sequencing data, and bulk RNA sequencing data and clinical data from The Cancer Genome Atlas

(TCGA), were used to shed light on epigenetic mechanisms and clinical consequences of oligodendroglioma evolution. The functional implications of these findings were validated in patients with newly diagnosed or recurrent oligodendroglioma using intraoperative subdural electrocorticography of tumor-infiltrated cortex, and a novel patient-derived organoid co-culture model of IDH-mutant glioma interactions with cortical neurons that was used for electrophysiology, calcium imaging, cell proliferation, IF, and pharmacologic experiments. In total, bioinformatic and imaging (n=25), human electrophysiological (n=11), and organoid functional data (n=17) were generated from 53 unique, non-overlapping patients.

*Nucleic acid extraction*

Genomic RNA and DNA were sequentially extracted from formalin-fixed, paraffin-embedded (FFPE) samples using the RNEasy FFPE Kit (Qiagen, 73504) and the QIAmp DNA FFPE Kit (Qiagen, 54604). Nucleic acid quantity and quality were assessed using a Nanodrop 8000 (ThermoFisher) and a TapeStation (Agilent Technologies).

*Spatial RNA sequencing and analysis*

Spatial transcriptomic profiling was performed on FFPE blocks with extracted RNA DV200% values greater than 50%, using the 10x Genomics Visium Spatial assay (10x Genomics, 1000336). 6mm cores were mounted within capture areas on Visium glass slides, deparaffinized, stained with hematoxylin and eosin (H&E), and imaged at the Gladstone Institutes Histology Core. Libraries were prepared according to manufacturer instructions at the Gladstone Institutes Genomics Core and sequenced on an Illumina NovaSeq 6000 or Novaseq X at the UCSF Center for Advanced Technology. Sequencing was performed with the recommended protocol (read 1: 28 cycles, i7 index read: 10 cycles, i5 index read: 10 cycles, read 2: 91 cycles). FASTQ sequencing files and histology images were processed using the 10x SpaceRanger pipeline and the Visium Human Transcriptome Probe Set v1.0 GRCh38-2020-A. Data were visualized using the 10x Loupe Browser software (v8.1) and Seurat R package (v4.3.0).

SpaceRanger generated filtered feature matrices were imported into a Seurat object (v4.3.0, arguments min.cells=3, min.features=100) using R (v4.2.1) and RStudio (v2022.07.2 Build 576). The individual count matrices were normalized based on nFeature_RNA count with less than 10% of reads attributed to mitochondrial transcripts. Dimensionality reduction was performed on the normalized filtered feature-barcode matrix using principal component analysis (PCA). Uniform manifold approximation and projection (UMAP) analysis and Louvain clustering were performed on the reduced data, followed by marker identification and differential gene expression. Parameters for downstream analysis included a minimum distance metric of 0.2 for UMAP, resolution of 0.25 for Louvain clustering as determined using clustree (v0.5.0), and a minimum difference in fraction of detection of 0.25 and a minimum log-fold change of 0.25 for marker identification. UMAP projections and cluster distributions were visualized in the Loupe browser as needed, after combining spatial transcriptomic data from individual capture areas using the 10x Spaceranger aggr pipeline (v2.0.0). Batch effects, which can confound spatial transcriptome analyses[29,31], were corrected using Harmony (v0.1.1) by iteratively varying the sigma and theta values to eliminate dataset-specific and technical differences while preserving biological differences. Differential expression analyses were performed using (1) mean gene expression in each spatial transcriptomic cluster, (2) $\log_2$ fold-change of gene mean expression in a spatial transcriptomic cluster relative to all other spatial transcriptomes, and (3) a p-value denoting gene expression significance in each spatial transcriptomic cluster relative to spatial transcriptomes in other clusters. P-values in each cluster were adjusted for false discovery rate to account for the number of genes being tested. Heatmaps of spatial transcriptomic data were generated using the DoHeatmap feature expression function in Seurat.

Histologic annotations were performed by a board-eligible neuropathologist (KM) on a spatial transcriptome-by-spatial transcriptome basis using the 10x Loupe Browser. Gene set enrichment analysis was performed on clusters using cell type signature gene sets from MSigDB (https://www.gsea-msigdb.org/gsea/msigdb) with the fgsea R package (Bioconductor v3.16). As previously described[29,30], copy number analysis within spatial transcriptomes was performed using the inferCNV (v1.14.0) and SpatialInferCNV R packages (v0.1.0). A cluster of distant normal cortex with no infiltrating tumor cells on H&E or IDH1 R132H IHC staining was designated as reference. All cluster annotations were exported into a csv file and imported into R along with the aggregate filtered feature matrix. The count matrix, annotated clusters and a gene order file were input into inferCNV (arguments were cutoff = 0.1, analysis_mode=subclusters, HMM = TRUE and denoise = TRUE) to generate a six-state copy number alteration probability model for each spatial transcriptomic cluster. Final cluster annotations were performed using a combination of cell signature gene sets, differentially expressed cluster marker genes, per spatial transcriptome information from H&E and IDH1 R132H IHC images, and presence or absence of chromosome 1p/19q whole arm codeletion.

Trajectory analyses were performed using monocle3 (v1.3.1) for pseudotime, and velocyto (v0.17.16) with scVelo (v0.2.5) for RNA velocity. For pseudotime analysis, data were normalized followed by UMAP dimensionality reduction as described above. The ‘cluster_cells’ and ‘learn_graph’ monocle commands were used with default parameters and cells were ordered along pseudotime after manually selecting a root node (based on cluster, cell type, and cell cycle information). For RNA velocity analysis, velocyto was used to generate loom files with spliced and unspliced mRNA count information. scVelo was used to filter and normalize gene expression using criteria “min_shared_counts=2’, and ‘n_top_genes=3000’ prior to computing RNA velocity and latent time. RNA velocity was visualized by projecting on to the UMAP generated using R and Seurat.

Deconvolution of spatial gene expression programs was performed using single-cell/single-nuclear IDH-mutant and IDH-wildtype glioma cell types[21,35]. To do so, a gene set of differentially expressed marker genes from the reference dataset was created. An average geneset expression score was calculated for each spatial transcriptomic spot using the AddModuleScore function in Seurat and visualized as feature plots projected on UMAP or the spatial tissue.

Gene set signature scoring for 28 genes associated with synaptic connectivity was performed using the AddModuleScore function in UCell (v2.11.1) which avoids population wide binning of gene expression and allows more uniform comparisons within and across different datasets. The score was visualized as feature plots using SCpubr (v2.0.2).

Gene ontology and gene set enrichment analyses were performed using the clusterProfiler R package (v3.2). Briefly, the 100 most differentially expressed genes were included in the comparison category of interest, and gene ontology over representation analysis was performed using the enrichGO function (pvalueCutoff = 0.01, qvalueCutoff = 0.05, "Biological Processes" ontology). Additional ontology domains such as Cellular Component (CC), Molecular Function (MF) were also interrogated as needed.

*Targeted next-generation DNA sequencing and analysis*

Targeted DNA sequencing was performed using the UCSF500 next-generation sequencing panel, as previously described[29]. In brief, this capture-based next-generation DNA sequencing assay targets all coding exons of 479 cancer-related genes, select introns, and upstream regulatory regions of 47 genes to enable detection of structural variants such as gene fusions and DNA segments at regular intervals along each chromosome to enable genome-wide copy number and zygosity analyses, with a total sequencing footprint of 2.8 Mb. Multiplex library preparation was performed using the KAPA Hyper Prep Kit (Roche, 07962355001,). Hybrid capture of pooled libraries was performed using a custom oligonucleotide library (Nimblegen SeqCap EZ Choice). Captured libraries were sequenced as paired-end reads on an Illumina NovaSeq 6000 at >200x coverage for each sample. Sequence reads were mapped to the reference human genome build GRCh37 (hg19) using the Burrows-Wheeler aligner (v0.7.17). Recalibration and deduplication of reads was performed using the Genome Analysis Toolkit (v4.3.0.0). Coverage and sequencing statistics were determined using Picard (v2.27.5), CalculateHsMetrics, and CollectInsertSizeMetrics. Single nucleotide variant and small insertion/deletion mutation calling was performed with FreeBayes, Unified Genotyper, and Pindel. Large insertion/deletion and structural alteration calling was performed with Delly. Variant annotation was performed with Annovar. Single nucleotide variants, insertions/deletions, and structural variants were visualized and verified using Integrative Genome Viewer (v2.16.0). Genome-wide copy number and zygosity analysis was performed by CNVkit and visualized using NxClinical (Biodiscovery, v6.0).

*Single-cell ATAC sequencing analysis*

Publicly available single cell Assay for Transposase Accessible Chromatin (ATAC) sequencing data were analyzed using Seurat (v4.3.0) and Signac (v1.14.0). Briefly, single cell matrices, filtered peak matrices, and fragment files were used to generate Seurat objects (min.cells = 3, min.features = 100). Gene annotation information was added, unified peaks were called for all oligodendroglioma cases, and datasets were merged using the standard Signac workflow. Transcription start site accessibility enrichment scores were calculated using the TSSEnrichment function (±1000 base pairs) for all protein coding genes and for the 28 genes associated with synaptic connectivity. The score was visualized using TSSPlot and grouped by CNS WHO grade of tumors. Individual coverage plots with gene annotation were generated for all 28 genes including genomic regions 2000 bp upstream of each gene. The plots included pseudobulked peak plots per gene as well as per gene-per cell fragment abundance.

*Histology, immunohistochemistry, immunofluorescence, and microscopy*

For adult human tissue samples, tissue was processed, embedded in paraffin, and 4μm FFPE tissue section H&E staining was performed using standard clinically validated procedures. IHC for IDH1 R132H (1:500, Dianova, DIA-H09-L), ATRX (1:100, SIGMA, polyclonal), Ki-67 (1:50, DAKO, MIB1) was performed with appropriate controls using a Leica Bond III platform. H&E and IHC sections were imaged on a Leica Aperio GT 450 microscope using a 40x objective. Images were obtained and analyzed using Aperio ImageScope software (v12.4.3.5008).

For IF staining of FFPE sections, 5μm sections underwent rehydration and deparaffinization, and were processed for antigen retrieval by heating in a commercially available solution (Invitrogen, 00-4955-58, antigen retrieval solution pH 6). Sections were quenched with 3% hydrogen peroxide (VWR Chemicals, BDH7540-2) to block endogenous peroxidase activity, permeabilized (1% goat serum and 0.4% Triton X-100), and blocked for non-specific binding (5% goat serum and 0.4% Triton X-100). Primary antibody incubations were performed overnight at 4°C using the following antibodies: chicken anti-NDRG2 (1:250, Aves lab, NDRG2-0020), mouse anti-TSP-1 (1:20, Invitrogen, MA5-13398), and rabbit anti-connexin 43 (1:400, Sigma Aldrich, C6219).

For IF staining of cortical neurospheres, glioma organoids, and co-culture experiments, samples were fixed in 4% paraformaldehyde (PFA) on a rocker for 45 minutes at 4°C, washed in PBS for 2 hours, and placed in 30% sucrose overnight at 4°C. Once samples sunk to the bottom of wells, they were placed in cryomolds and sectioned at 12μm thickness. Sections were washed with PBS for 15 minutes, blocked for non-specific binding in 10% normal goat serum (NGS) in PBS for 1 hour at room temperature, and incubated with buffer solution (5% NGS and 0.25% Triton-X in PBS). Primary antibody incubations were performed overnight at 4°C using rabbit anti-Ki67 (1:500, Abcam, ab15580), mouse anti-human nuclear antigen (HNA, 1:100, Sigma-Aldrich, MAB1281), chicken anti-MAP2 (1:500, EnCor, CPCA-MAP2), mouse anti-Synapsin1 (1:200, Invitrogen, MA5-31919), and rabbit anti-Homer1 (1:250, Invitrogen, PA5-21487). Slides were washed three times in fresh PBS for 15 minutes per wash using a slide mailer box. Secondary antibody solutions were prepared in buffer containing 1% NGS and 0.25% Triton X-100 in PBS. Secondary antibody incubations were performed for 1 hour at room temperature using Alexa Fluor 488 goat anti-chicken IgY (H+L) (1:500, Invitrogen, A11039), Alexa Fluor 568 goat anti-mouse IgG (H+L) (1:500, Invitrogen, A11004), and Alexa Fluor 647 goat anti-rabbit IgG (H+L) (1:500, Invitrogen, A21245). Slides were washed 3 times in PBS, counterstained with DAPI (1:1000, Thermo-Fisher, 62248), mounted with Fluoromount-G mounting medium (SouthernBiotech, 0100-01) and imaged.

Fluorescent live-cell calcium imaging was performed using Fluo-4 AM dye (Invitrogen, F14201). Fluo-4 AM stock solution was prepared by adding 44μL of DMSO to one vial of Fluo-4 AM (50μg), followed by thorough vortexing. The stock solution (~860μM Fluo-4 AM in DMSO) was stored protected from light at -20°C, desiccated, and used within one week. For working solution preparation, 50μL of the Fluo-4 AM

stock was mixed with 14.3mL of fresh neuronal culture medium. Cultures were transferred to 24-well plates and incubated with 1mL of Fluo-4 AM loading solution per well in the dark at 37°C for approximately 60 minutes. Following incubation, cultures were washed 3 times with 1mL of fresh neuronal culture medium for 10 minutes per wash. After the final wash, cultures were transferred back into conditioned medium prior to imaging.

Confocal imaging was performed using a Nikon Eclipse Ti2-E inverted microscope. For imaging synaptic markers (Homer1, Synapsin1) as well as for organoid proliferation and invasion assays, images were acquired at 40x or 10x magnification at a resolution of 2048 x 2048 pixels. The laser dwell time was set to 2μs per pixel, and line averaging was performed once (Homer1, Synapsin1) or twice (organoid proliferation and invasion assays) to enhance the signal-to-noise ratio. Two-photon microscopy for calcium imaging was performed using a Nikon A1R two-photon microscope equipped with a 25x water immersion objective. The excitation wavelength was set at 920nm. Time-lapse imaging was conducted over 90 seconds with a frame acquisition rate of 512ms per frame. All imaging conditions, including laser power, gain, and scan speed, were optimized to balance temporal resolution with minimal photobleaching of samples.

For each region of interest in multiplexed IF staining, the total number of cells and the number of cells expressing different markers of interest were counted semi-automatically using Nikon NIS-Elements software (v5.42.05). Organoid proliferation was assayed by analyzing regions of interest (ROIs) using the FIJI Cell Counter plugin. Ki67-positive cells were identified and colocalized with HNA-positive cells to only include proliferating human patient tumor cells in quantitative analyses. The percentage of Ki67-positive cells was calculated by dividing the number of $Ki67^{+}$ $HNA^{+}$ double-positive cells by the total number of HNA-positive cells within each ROI. GliO-to-cNS invasion was assessed by imaging entire organoids and quantifying the number of HNA-positive cells invading the surrounding HNA-negative cNS. This quantification was performed using a custom script developed in QuPath. Synaptic marker analyses (Homer1 and Synapsin1) were performed by quantifying colocalized puncta using SynBot, a FIJI macro optimized for automated detection and analysis of synaptic marker colocalization[71]. The macro was manually parameterized to account for accurate signal intensity thresholds. Cell counts were obtained using QuPath, which was then used to divide by the number of colocalized puncta in each region. Post-acquisition analysis of IF images was performed using the Nikon NIS-Elements software (v5.42.05), FIJI (v2.9.0), and Photoshop (v26.2.0). Post-acquisition analysis of calcium dynamics was performed using FluoroSNNAP[72] adapted to run on MATLAB 2024b.

*Human electrocorticography and resting state local power-based connectivity analyses*

Electrocorticography (ECoG) recordings were performed intraoperatively under clinical indications, prior to tumor resection, in eight patients with newly diagnosed and two patients with recurrent CNS WHO grade 2 oligodendroglioma, IDH-mutant, and 1p/19q-codeleted, as previously described[22,73]. Briefly, resting state ECoG signals were obtained from subdural arrays after an average anesthesia washout of 20 minutes and a wakefulness test[22,73] to confirm that patients had returned to their cognitive baseline.

As recordings were obtained, an initial 180 second resting period was acquired at 4,800 Hz, during which participants were asked to close their eyes and not speak. Each patient's recording was down-sampled to 1,200 Hz, and channels with excessive noise artifacts (kurtosis exceeded 5.0) were visually identified and removed. The remaining channels were referenced to a common average. To remove slow drift artifacts, recordings were high-pass filtered at 0.1 Hz. Morlet wavelets were constructed by convolving a complex sine wave with a Gaussian, isolate the high-gamma power (HGP) band range (center frequencies ranging from 70 to 150 Hz with equal length wavelets across each frequency). To compute each electrode's local HGP connectivity with respect to adjacent electrodes, sliding windows of 5 seconds with a 2.5-second step were created from the 180-second resting-state recording. Spearman correlations in HGP were performed between each electrode pair for each window, accounting for time-lagged interactions within a range of ±50ms. The correlation values for each lag were aggregated across windows, and the lag with the maximum median correlation was identified for each electrode pair. The correlation score per window was averaged to provide each electrode's pairwise average connectivity score. The median of each electrode's pairwise connectivity scores yielded a single HGP connectivity score per electrode. All electrodes in the study cohort were pooled, and a Student's t-test was used to compare electrode-level HGP connectivity between patients with newly diagnosed or recurrent oligodendroglioma.

*LASSO and Elastic Net regression*

Gene expression risk score development and validation were performed using data from The Cancer Genome Atlas (TCGA) pan-cancer atlas (https://gdc.cancer.gov/about-data/publications/pancanatlas)[34]. Gene expression data were obtained using the RNA Sequencing dataset (EBPlusPlusAdjustPANCAN_IlluminaHiSeq_RNASeqV2.geneExp.tsv). Only newly diagnosed tumor samples were included by filtering TCGA Biospecimen Core Resource (BCR) barcodes for sample numbers containing the "01" and "03" designator. Clinical information was obtained from the TCGA-Clinical Data Resource (CDR) Outcome Dataset (TCGA-CDR-SupplementalTableS1.xlsx) and was matched to copy number alteration (CNA) data by BCR barcode. DNA mutation data was obtained from the Mutations dataset (mc3.v0.2.8.PUBLIC.maf.gz) with the *maftools* (v2.18.0) package in R. CNAs were determined using the Copy Number Dataset (broad.mit.edu_PANCAN_Genome_Wide_SNP_6_whitelisted.seg). Chromosome segments with mean intensity values less than -0.1 were defined as lost, and mean intensity values greater than +0.15 were defined as gained, as previously described[74]. IDH-mutant gliomas were identified within the "GBM" or "LGG" tumor type identifier, by filtering for tumors with *IDH1* or *IDH2* mutation. Tumors were further stratified into either astrocytoma or oligodendroglioma based on codeletion of chromosomes 1p and 19q, which was defined as a cumulative loss of at least 95% of the length of each chromosome arm.

The resulting cohort was split into training and testing sets at a ratio of 0.75 to 0.25. $Log_2$-transformed RNA sequencing gene expression values were used to train LASSO and Elastic Net regularized Cox regression models to predict progression-free or overall survival with the concordance index (c-index) for each target endpoint, using the *glmnet* and *cv.glmnet* functions from the *glmnet* package (v4.1-8) in R, as previously described[75]. Elastic Net model selection was performed by selecting an optimal alpha value from a range of 0.05 to 0.95. Model training was performed using 10-fold cross validation. Predicted risk values for each model were linearly rescaled from 0 to 1 using the maximum and minimum values in the training set, and model performance was measured using c-index in univariable Cox proportional hazards models in the testing set using the *survival* (v3.7-0) package in R. Risk scores were then divided using the maximally selected rank statistic into two risk groups (low connectivity or high connectivity) using the *surv_cutpoint* function from the *survminer* (v0.5.0) package in R, and risk stratification was measured using the Kaplan Meier method and log-rank tests.

*Organoid creation and glioma-neuron co-culture experiments*

Oligodendroglioma (GliO) and astrocytoma (GliA) glioma organoids were generated as previously described[63] . In brief, freshly resected tumor tissue from patients with known or suspected oligodendroglioma or astrocytoma was transferred from the operating room to the laboratory on ice with a target cold ischemia time of under 30 minutes. Tumor tissue was processed immediately or suspended in Hibernate A (Gibco, A1247501) for up to 6 hours post-resection. Subsequently, ~2-3mm$^3$ tumor fragments were incubated with RBC lysis buffer (Invitrogen, 00-4333-57) for 10 minutes at room temperature with gentle rocking to remove contaminating red blood cells. Tumor fragments were washed once in media containing Hibernate A supplemented with 1:100 Glutamax (Gibco, A1286001) and 1:100 Anti-Anti (Gibco, 15240062). Tumor fragments were then washed twice in culture media containing a 1:1 mixture of Neurobasal Medium (Gibco, 21103049) and DMEM/F12 (Gibco, 11320033), supplemented with 1:50 B-27 without Vitamin A (Gibco, 12587001), 1:100 N-2 (Gibco, 17502001), 1:100 Glutamax, 1:100 Penicillin-Streptomycin (Gibco, 15140122), low-glutamate non-essential amino acids mixture (Gly, L-Ala, L-Asn, L-Asp, L-Pro, and L-Ser at 100µM and L-Glutamic acid and 300nM), 0.05mM 2-mercaptoethanol (Sigma-Aldrich, 63689), and 2.5µg/mL insulin (Sigma-Aldrich, I9278). Tumor fragments were then cut using 1mm biopsy punches and transferred to 24-well ultra-low attachment plates containing 1mL fresh culture medium per well. The resulting glioma organoids were incubated in a humidified hypoxic sterile incubator on an orbital shaker at 120RPM and 37°C, with 5% $CO_2$ and 5% $O_2$. Culture media were refreshed every other day, and organoids were cultured for a minimum of 4 weeks prior to experimentation. Mycoplasma contamination was tested at regular intervals using the PCR-based MycoAlert kit (Lonza, LT07-318).

Cortical neurospheres (cNS) were isolated as previously described[22]. In brief, primary cortical cultures were established using C57BL/6J E15.5-18.5 embryos from timed-pregnant dams that were sacrificed in accordance with UCSF Institutional Animal Care and Use Committee (IACUC) guidelines using $CO_2$ euthanasia. The complete cortex of E15.5-18.5 embryos was dissected in ice-cold Neurobasal medium (Gibco, 21103049) under a Carl Zeiss dissecting microscope. Dissected cortices were minced into 1mm$^2$ pieces and enzymatically digested using the Worthington Papain Dissociation System (Worthington Biochemical Corporation, LK003150), with tissue incubation in papain solution for 7 minutes followed by papain inhibition for 3 minutes at 37°C with constant agitation. The inhibitor was then removed, and 5ml of Neurobasal medium supplemented with 0.5ml of 10 mg/ml DNase (Worthington, LK003172) was added for the last step of enzymatic dissociation, followed by centrifuged for 6 minutes at 4°C. The supernatant was then discarded, and fresh Neurobasal media was added. The suspension was filtered through a 40μm nylon mesh into a new 50mL conical to remove large tissue fragments. The filtered suspension was centrifuged at 1400 RPM for 6 minutes at 4°C, and the pellet was resuspended in pre-warmed neuronal culture medium containing Neurobasal medium supplemented with 1:100 N-2, 1:100 Anti-Anti, 1:50 B-27 Supplement, and 1:100 Sodium Pyruvate (Gibco, 11360070). Cell viability and density were assessed by mixing the cell suspension with trypan blue and counting live cells using a hemocytometer. Cells were plated at a density of 500,000 cells per well in a 24-well ultra-low attachment plate containing 1mL of neuronal culture medium. Plates were then incubated on an orbital shaker at 120 RPM in a humidified hypoxic sterile incubator at 37°C with 5% $CO_2$, and 5% $O_2$. Half-media changes were performed every other day.

GliO or GliA co-culture fusions with cNS were performed by placing a single glioma organoid and a single 4 days in vitro (DIV) cNS in a single well containing 1mL neuronal culture media in a hypoxic incubator at a 45°angle for a minimum of 48 hours. Once the cultures were attached, plates were put back onto a 120 RPM orbital shaker in the incubator. All experiments were performed 21 days post-culture.

Seventeen patient-derived oligodendroglioma or astrocytoma cell cultures were collected and used for the experiments in this study. All experiments were performed in biological duplicates or triplicates and used the following cell cultures: SF0567, SF0556, SF0580, SF0606, SF0609, SF0616, SF0620, SF0621, SF0623, SF0624, SF0625, SF0632, SF0638, SF0642, SF0647, SF0649, and SF0653.

*Multielectrode array experiments*

24-well CytoView multi-electrode array plates (Axion Biosystems, M384-tMEA-24W, 11-01.03.00387), containing 4 x 4 electrode grids with 16 channels spaced 350μm apart were used for multi-electrode array (MEA) data acquisition. Each well was coated with 100μL Poly-D-Lysine (Gibco, A3890401) 2 days prior to organoid transfer and incubated overnight in a hypoxic incubator at 37°C with 5% $CO_2$, and 5% $O_2$. After incubation, the Poly-D-Lysine solution was removed, and wells were rinsed twice with ultrapure distilled water. One day prior to organoid transfer, MEA plates were coated with 100μL per well 10μg/mL laminin mouse protein solution (Corning, CB-40232) in PBS and incubated overnight in a hypoxic incubator at 37°C with 5% $CO_2$, and 5% $O_2$.

cNS or glioma organoid plus cNS co-cultures chosen for MEA plating were at least 10 DIV. Immediately before organoid transfers, laminin coating solution was aspirated without rinsing. Organoids were plated in neuronal culture medium, with 1 cNS or 1 co-culture per well in 1mL of fresh medium per well of the 24-well plate. MEA plates were placed on an isolated shelf in a hypoxic incubator at 37°C with 5% CO2, and 5% O2. Plates were left undisturbed for 48 hours to allow organoids to attach, with no media changes performed during this period.

Spontaneous extracellular electrical recordings were collected using the Axion Biosystems Maestro Edge system, as previously described[22]. MEA plates were maintained on a heated stage at 37°C and ventilated with a mixture of 5% $CO_2$ and 95% ambient air for the duration of 15-minute recordings. Electrical (spike) events were detected using an adaptive threshold-crossing method, wherein the spike detection threshold was set to 5 standard deviations above the root mean square (RMS) noise level for each electrode. A well was considered active if 4 or more electrodes detected electrical activity. Data were acquired with a 200-3,000Hz bandpass filter, 1000x gain, and 12.5kHz sampling rate per channel.

Raw MEA data were processed using the Statistics Compiler function in AxIS to calculate weighted mean firing rate (the mean firing rate based only on electrodes with activity greater than the minimum spike rate of 5 spikes per minute, in Hz), burst frequency (the total number of single-electrode bursts divided by the duration of the analysis, which was 15 minutes, in Hz), burst percentage (the number of spikes in single-electrode bursts divided by the total number of spikes, multiplied by 100), and synchronization index (a unitless measure reflecting the degree of synchronicity within the neuronal network, scaled between 0 and 1, which was quantified using the area under the normalized cross-correlogram, with values closer to 1 indicating greater synchrony)[76]. All MEA statistics were normalized to the mean of the control group (cNS conditions).

*Drug Treatment*

GliO + cNS co-culture fusions were treated with two doses of 50μM gabapentin or 50μM meclofenamate, 48 hours apart, as previously described[22,26,77]. MEA recordings were performed daily, and co-cultures were fixed after 96 hours of treatment for downstream proliferation analyses.

*Statistics*

All experiments were performed with independent biological replicates and repeated, and statistics were derived from biological replicates. Biological replicates are indicated in each figure panel or figure legend. No statistical methods were used to predetermine sample sizes, but sample sizes in this study are similar or larger to those reported in previous publications. Data distribution was assumed to be normal, but this was not formally tested. Investigators were blinded to conditions during clinical data collection and analysis of mechanistic or functional studies. Bioinformatic analyses were performed blind to clinical features, outcomes or molecular characteristics. The clinical samples used in this study were retrospective and nonrandomized with no intervention, and all samples were interrogated equally. Thus, controlling for covariates among clinical samples is not relevant. Cells and organoids were randomized to experimental conditions. No clinical, molecular, or cellular data points were excluded from the analyses. Unless specified otherwise, lines represent means, and error bars represent standard error of the means. Results were compared using Student's t-tests and other statistical approach, which are indicated in figure legends alongside approaches used to adjust for multiple comparisons. In general, statistical significance is shown by asterisks (*p£0.05, **p£0.01, ***p£0.0001), but exact p-values are provided in the figure legends when possible.

*Reporting summary*

Further information on research design is available in the Nature Research Reporting Summary linked to this article.

#### Methods-only references

68. Drexler, R. *et al.* A prognostic neural epigenetic signature in high-grade glioma. *Nat. Med.***30**, 1622–1635 (2024).
69. Harwood, D. S. L. *et al.* Glioblastoma cells increase expression of notch signaling and synaptic genes within infiltrated brain tissue. *Nat. Commun.***15**, 7857 (2024).
70. Lee, S. *et al.* High-throughput identification of repurposable neuroactive drugs with potent anti-glioblastoma activity. *Nat. Med.***30**, 3196–3208 (2024).
71. Savage, J. T. *et al.* SynBot is an open-source image analysis software for automated quantification of synapses. *Cell Rep. Methods***4**, 100861 (2024).
72. Patel, T. P., Man, K., Firestein, B. L. & Meaney, D. F. Automated quantification of neuronal networks and single-cell calcium dynamics using calcium imaging. *J. Neurosci. Methods***243**, 26–38 (2015).
73. Aabedi, A. A. *et al.* Functional alterations in cortical processing of speech in glioma-infiltrated cortex. *Proc. Natl. Acad. Sci.***118**, e2108959118 (2021).
74. Choudhury, A. *et al.* Meningioma DNA methylation groups identify biological drivers and therapeutic vulnerabilities. *Nat. Genet.***54**, 649–659 (2022).
75. Chen, W. C. *et al.* Targeted gene expression profiling predicts meningioma outcomes and radiotherapy responses. *Nat. Med.* 1–1 (2023) doi:10.1038/s41591-023-02586-z.
76. Paiva, A. R. C., Park, I. & Príncipe, J. C. A comparison of binless spike train measures. *Neural Comput. Appl.***19**, 405–419 (2010).
77. Hausmann, D. *et al.* Autonomous rhythmic activity in glioma networks drives brain tumour growth. *Nature***613**, 179–186 (2023).

## Figures

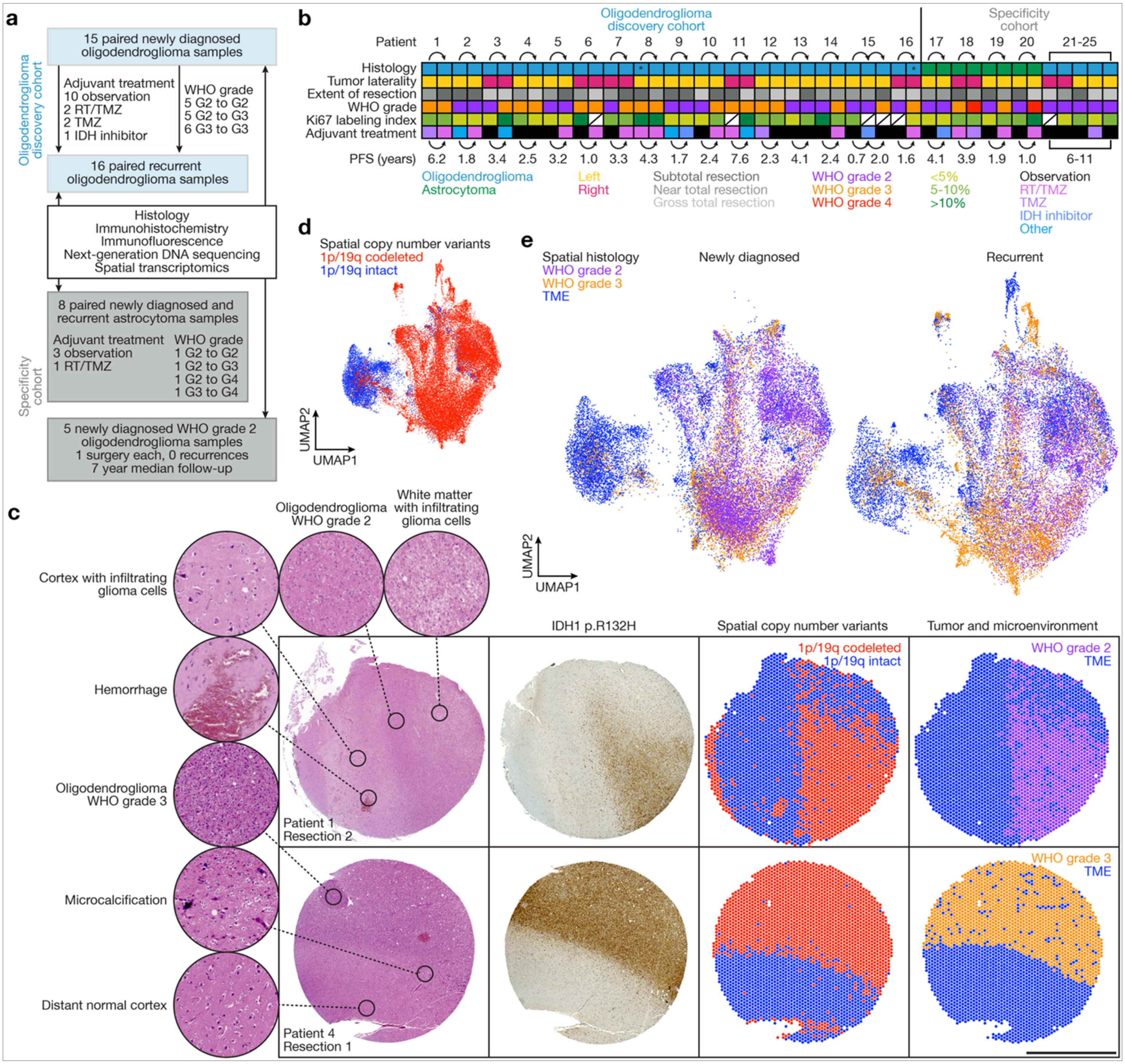

### Figure 1

**Patients and workflow for spatial sequencing of serial IDH-mutant diffuse gliomas samples. a**, 31 patient-matched newly diagnosed and recurrent oligodendroglioma samples from 16 patients, 8 patient-matched newly diagnosed and recurrent astrocytoma samples from 4 patients, and 5 newly diagnosed oligodendroglioma samples from 5 patients without documented recurrence that were analyzed using histological approaches, immunohistochemistry, immunofluorescence, targeted next-generation DNA sequencing, and spatial RNA sequencing. **b**, Oncoplot showing clinical, histopathological, and genetic information from the new IDH-mutant diffuse glioma samples reported in this study. Each column represents a separate tumor resection, and the adjuvant treatment row shows the postoperative therapies each patient received after the resection in the corresponding column. Samples that did not pass quality control for spatial RNA sequencing are marked with an asterisk. **c**, Histopathological annotation of the IDH-mutant diffuse glioma samples included in this study plus IDH1 R132H immunohistochemistry and spatial copy number analyses that were used to distinguish tumor from tumor microenvironment (TME) spatial transcriptomes. Scale bar, 1mm. **d**, Uniform manifold approximation and projection (UMAP) of 55,737 oligodendroglioma spatial transcriptomes after pre-processing and Harmonization, shaded by presence or absence of chromosome 1p/19q-codeletion. **e**, UMAP shaded by CNS WHO grade of oligodendroglioma spatial transcriptomes versus non-tumor microenvironment spatial transcriptomes.

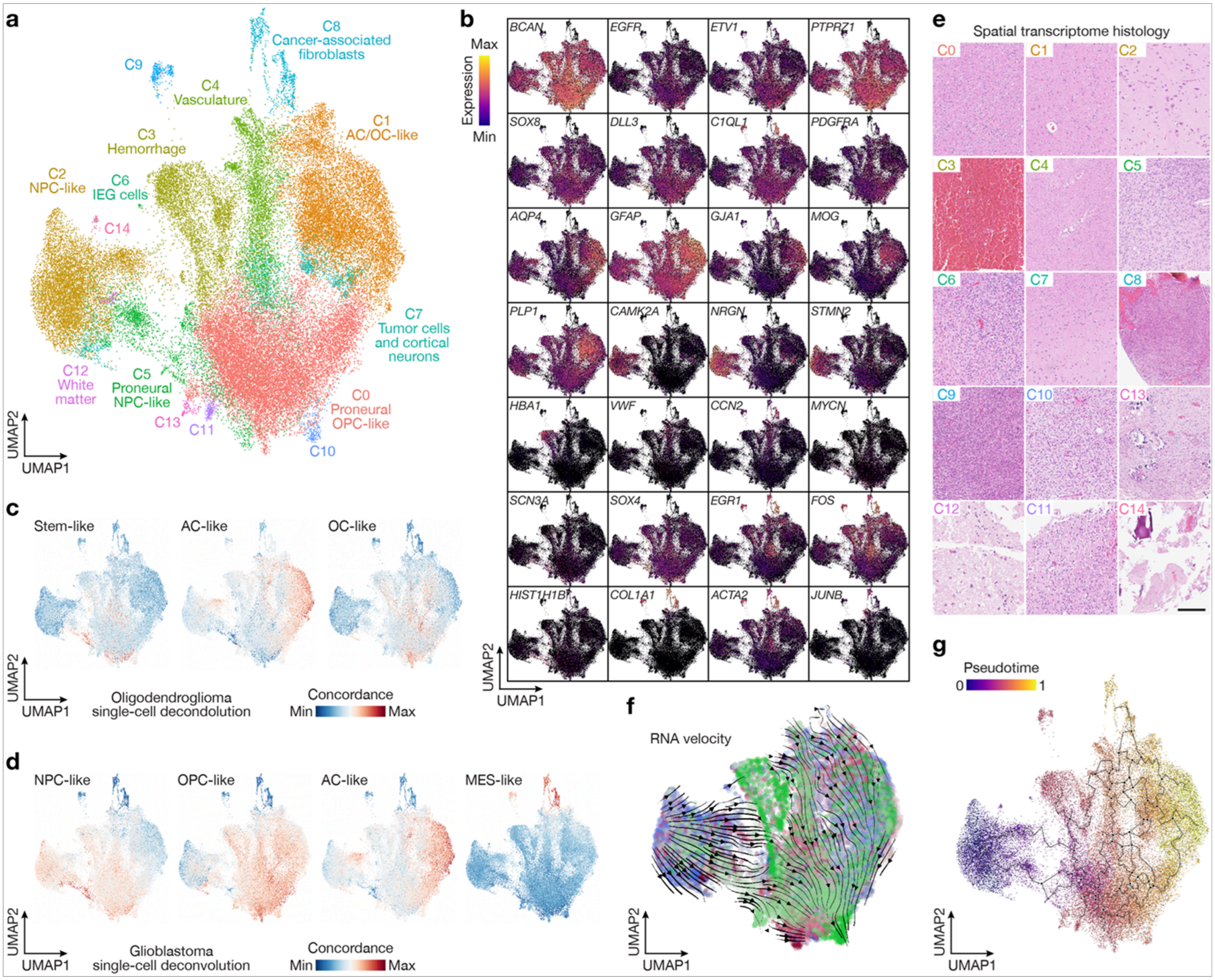

### Figure 2

**Oligodendroglioma spatial transcriptomes evolve from NPC-like and proneural NPC-like cell states. a**, Spatial RNA sequencing UMAP of 55,737 transcriptomes from 31 oligodendroglioma samples showing tumor cell states and microenvironment cell types. **b**, Feature plots showing differentially expressed marker genes across oligodendroglioma spatial transcriptome clusters. **c**, Feature plots showing deconvolved oligodendroglioma single-cell cell states from oligodendroglioma spatial transcriptomes. **d**, Feature plots showing deconvolved high-grade IDH-wildtype glioblastoma single-cell states from oligodendroglioma spatial transcriptomes. **e**, H&E stained images showing the most representative histology for each spatial transcriptomic cluster. Scale bar, 200μm. **f**, RNA velocity trajectory analysis of oligodendroglioma spatial transcriptomes. **g**, Pseudotime trajectory analysis of oligodendroglioma spatial transcriptomes.

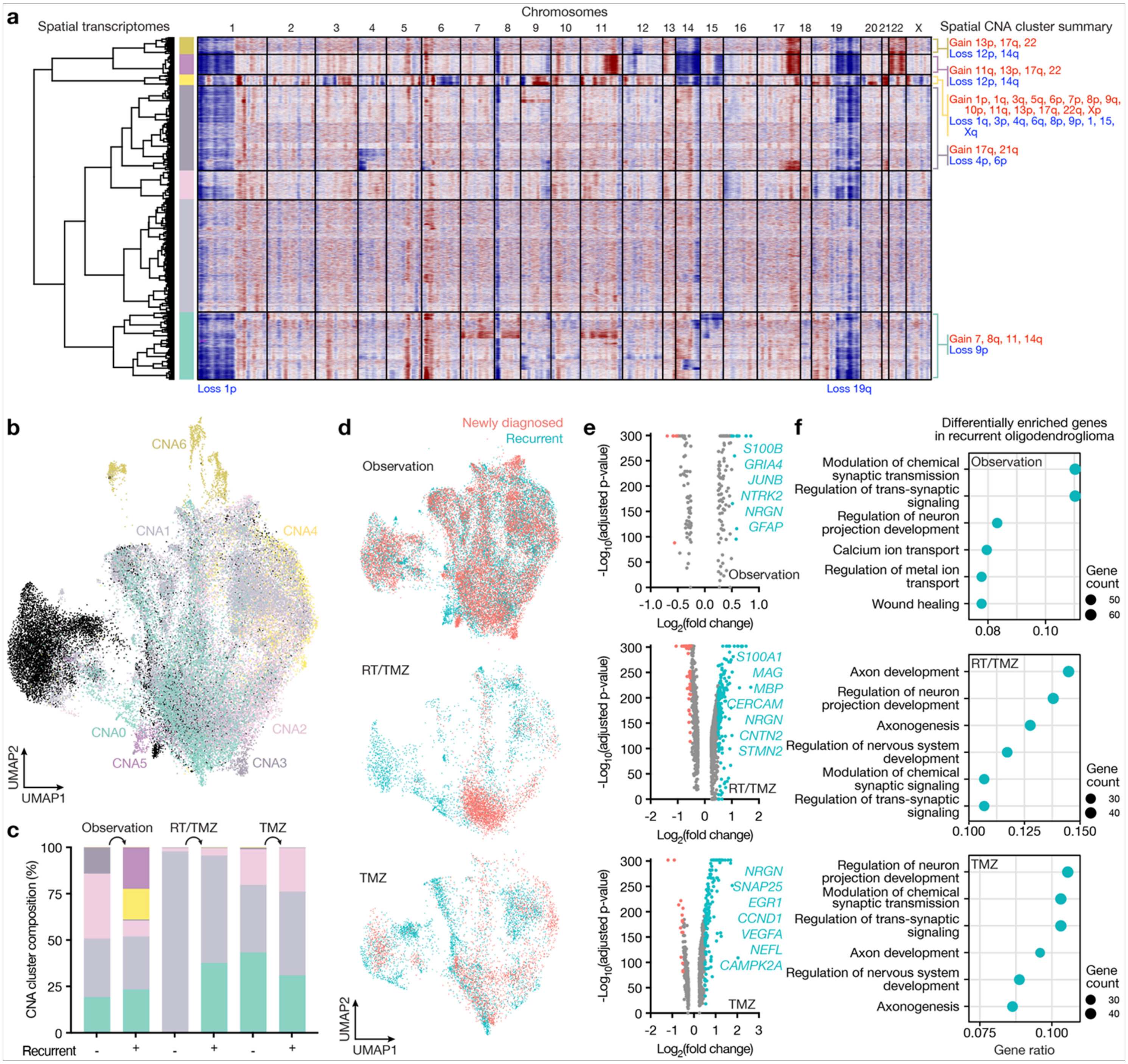

### Figure 3

**Recurrent oligodendrogliomas are enriched in synaptic gene expression programs irrespective of previous therapy. a**, Heatmap visualization of unsupervised spatial copy number alteration (CNA) clusters across 31 oligodendroglioma samples. **b**, Oligodendroglioma spatial RNA sequencing UMAP shaded by CNA clusters. Spatial transcriptomes without CNAs from the tumor microenvironment are shown in black. **c**, Stacked bar plots showing relative distribution of CNA clusters for oligodendrogliomas that underwent observation (n=10), radiotherapy and temozolomide (RT/TMZ, n=2), or temozolomide only (n=2) between resections. **d**, UMAPs showing newly diagnosed and recurrent oligodendroglioma spatial transcriptomes according to therapies between resections. **e**, Volcano plots showing differentially expressed genes between recurrent and newly diagnosed oligodendroglioma spatial transcriptomes across adjuvant therapies. **f**, Gene ontology programs enriched in recurrent oligodendroglioma spatial transcriptomes across adjuvant therapies.

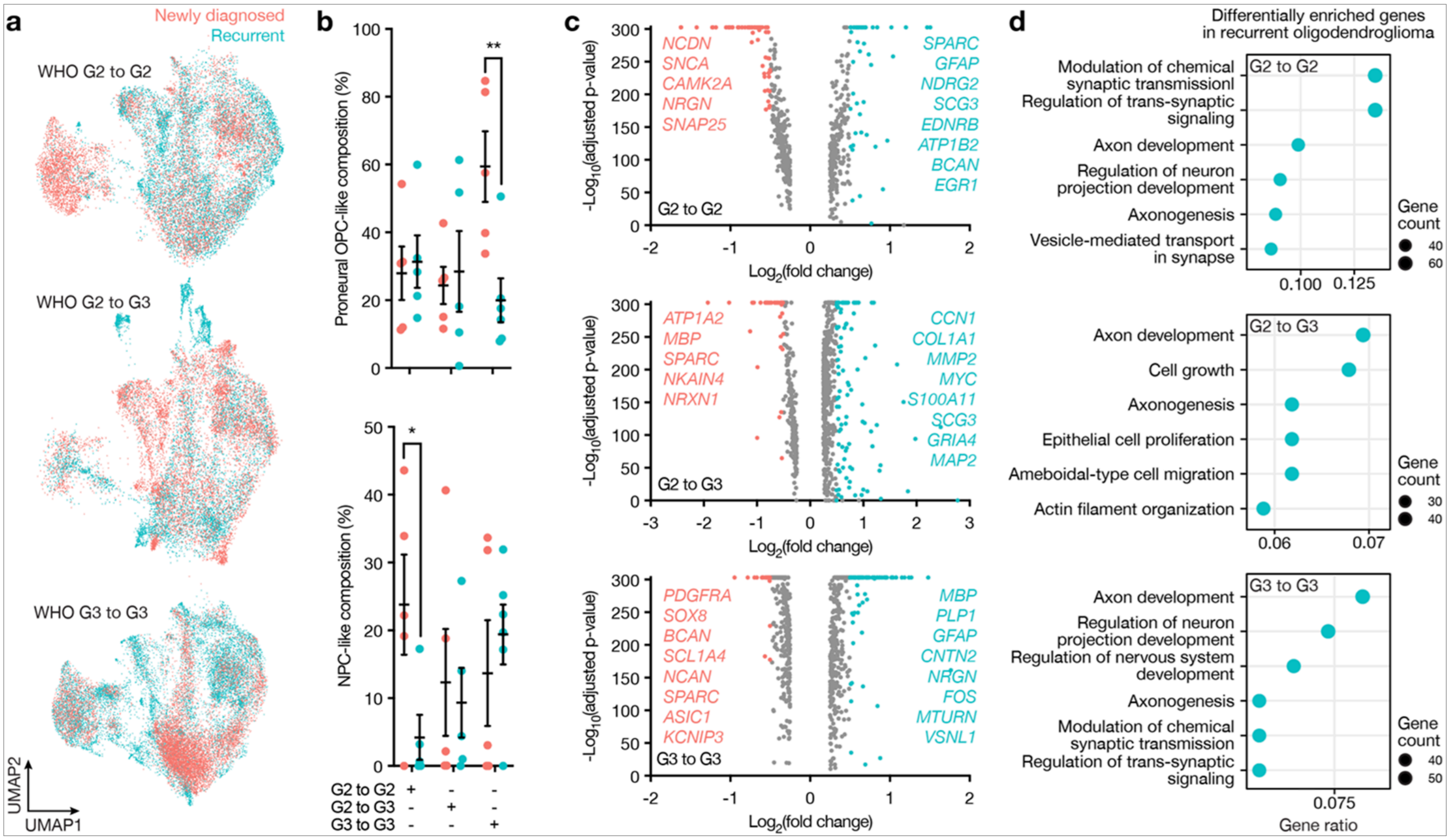

### Figure 4

**Recurrent oligodendrogliomas are enriched in synaptic gene expression programs irrespective of histopathologic grade. a**, UMAPs showing newly diagnosed and recurrent oligodendroglioma spatial transcriptomes according to CNS WHO grade. **b**, Quantification of Proneural OPC-like and NPC-like transcriptomes across newly diagnosed versus recurrent patient-matched oligodendroglioma spatial transcriptomes. Lines represent means, and error bars represent the standard error of the means. Student's t-tests, *p≤0.05, **p≤0.01. **c**, Volcano plots showing differentially expressed genes between newly diagnosed and recurrent oligodendroglioma spatial transcriptomes across CNS WHO grades. **d**,

Gene ontology programs enriched in recurrent oligodendroglioma spatial transcriptomes across CNS WHO grades.

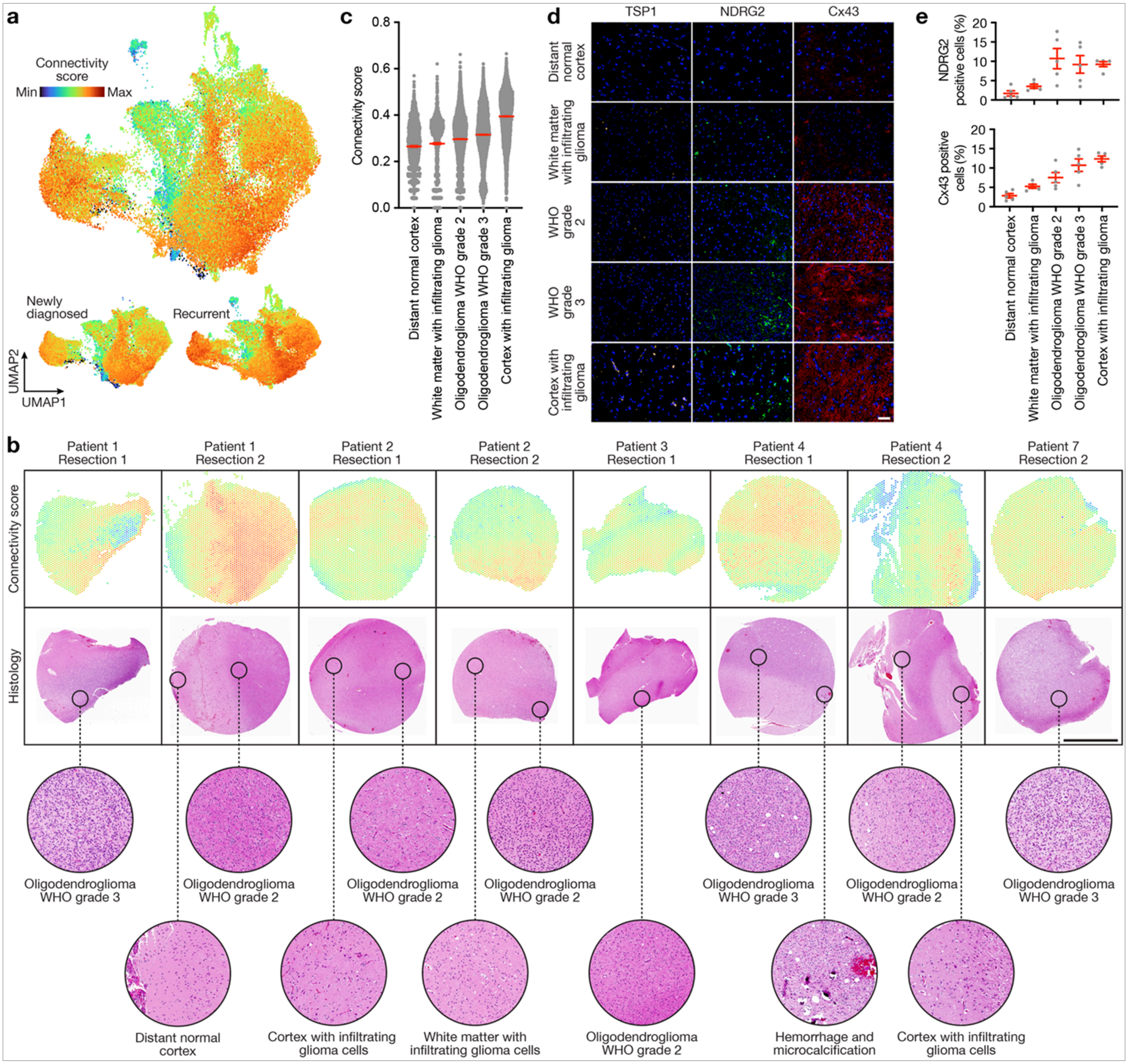

### Figure 5

**Synaptic gene expression programs underly oligodendroglioma evolution. a**, UMAP showing oligodendroglioma connectivity score derived from averaged expression of 28 differentially expressed genes in oligodendroglioma spatial transcriptomes from all tumors (top) or newly diagnosed versus recurrent tumors (bottom). **b**, Spatial connectivity score projection plus histopathological analysis of oligodendroglioma samples. **c**, Violin plots showing quantification of the connectivity score across distant normal cortex, white matter with infiltrating oligodendroglioma, densely cellular oligodendroglioma (CNS WHO grade 2 or 3), and cortex with infiltrating oligodendroglioma. Lines represent means and error bars represent the standard error of the means. One-way ANOVA, p<0.0001. **d**, Immunofluorescent staining on adjacent formalin fixed paraffin embedded tissue sections showing localization and distribution of TSP1 (*THBS1*), NDRG2, and Connexin 43 (*GJA1*), each of which are found in the 28-gene spatial connectivity score. Scale bar, 100µm.**e**, Quantification of NDRG2 and Connexin 43 immunofluorescence across distant normal cortex, white matter with infiltrating oligodendroglioma, densely cellular oligodendroglioma (CNS WHO grade 2 or 3), and cortex with infiltrating oligodendroglioma. Lines represent means and error bars represent the standard error of the means. One-way ANOVA, p<0.0001.

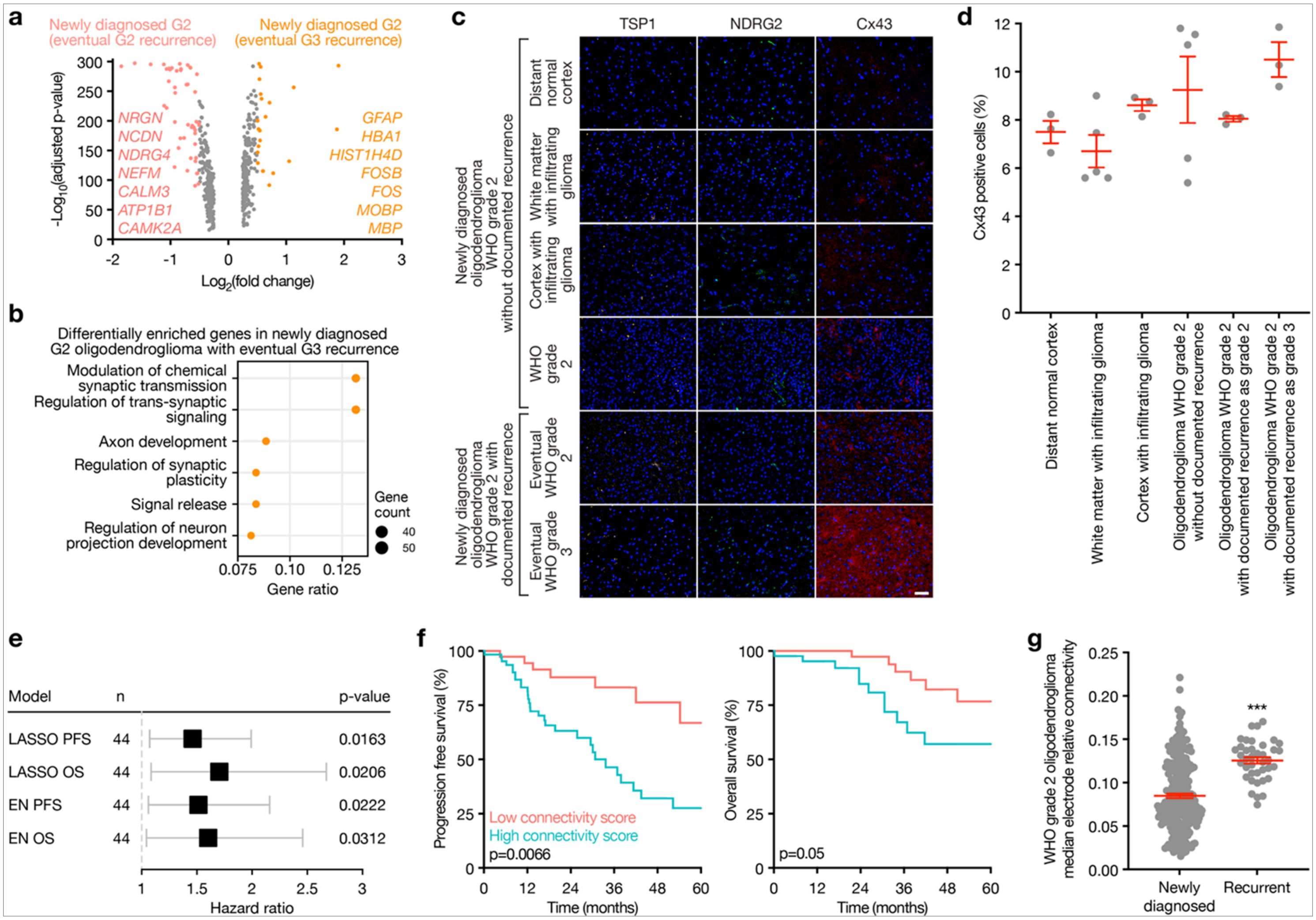

### Figure 6

**Synaptic gene expression programs and electrical connectivity underlie oligodendroglioma clinical outcomes. a**, Volcano plot showing differentially expressed genes in newly diagnosed CNS WHO grade 2 oligodendroglioma spatial transcriptomes between tumors with eventual recurrence as CNS WHO grade 3 (n=5) versus tumors with eventual recurrence that remained CNS WHO grade 2 (n=5). **b**, Gene ontology programs enriched in newly diagnosed CNS WHO grade 2 oligodendroglioma spatial transcriptomes that progressed to CNS WHO grade 3 at the time of recurrence. **c**, Immunofluorescent staining on adjacent formalin fixed paraffin embedded tissue sections showing localization and distribution of TSP1 (*THBS1*),

NDRG2, and connexin 43 (GJA1). Scale bar, 100μm. **d**, Quantification of Connexin 43 immunofluorescence across distant normal cortex, white matter with infiltrating oligodendroglioma, cortex with infiltrating oligodendroglioma, and densely cellular oligodendroglioma CNS WHO grade 2 without documented recurrence versus newly diagnosed densely cellular oligodendroglioma CNS WHO grade 2 with documented recurrence as grade 2 or grade 3. Lines represent means and error bars represent the standard error of the means. **e**, LASSO or Elastic Net (EN) regularized Cox regression hazard ratios for progression free survival (PFS) or overall survival (OS) per 0.1 increase in the spatial connectivity score in newly diagnosed oligodendrogliomas from The Cancer Genome Atlas (TCGA). Lines represent 95% confidence intervals. **f**, Kaplan Meier survival analyses for newly diagnosed IDH-mutant TCGA gliomas (n=109) based on low versus high spatial connectivity score. Long rank tests. **g**, Subdural electrode array relative connectivity from CNS WHO grade 2 oligodendrogliomas from 8 patients with newly diagnosed tumors (n=252 electrodes) and 2 patients with recurrent tumors (n=39 electrodes). Lines represent means and error bars represent the standard error of the means. Student's t text, ***p<0.0001.

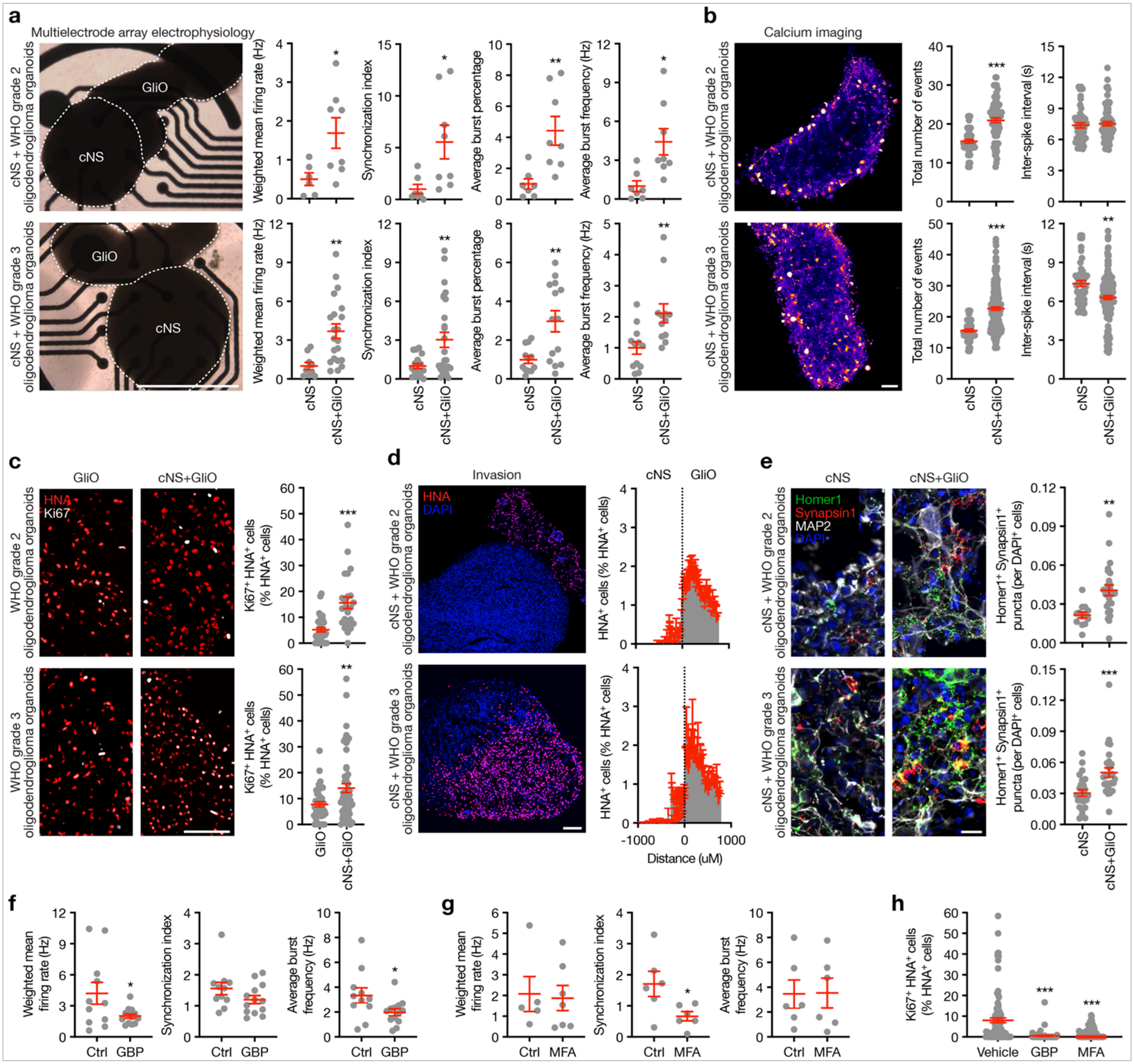

### Figure 7

**Patient-derived oligodendroglioma organoid co-cultures form electrical connections with neurons that drive increased tumor cell proliferation. a**, Representative bright-field microscopy images from multielectrode array (MEA) cultures of cortical neurospheres (cNS) alone or co-cultured with CNS WHO grade 2 or 3 oligodendroglioma patient-derived organoids (GliO). Weighted mean firing rate in Hertz (Hz), synchronization index, average burst percentage, and average burst frequency (Hz) are quantified. Top: n=7 cNS and n=8 cNS + CNS WHO grade 2 GliO co-cultures from two patients. Bottom: n=18 cNS and n=28 cNS + CNS WHO grade 3 GliO co-cultures from four patients. Scale bar, 1000µm. **b**, Representative maximum projection images from two-photon calcium microscopy of cNS alone or cNS + CNS WHO

grade 2 or 3 GliO co-cultures. Total calcium events and inter-spike interval in seconds (s) are quantified. Top: n=48 cells from 3 cNS and n=71 cells from 4 cNS + CNS WHO Grade 2 GliO co-cultures. Bottom: n=48 cells from 3 cNS and n=183 cells from 6 cNS + CNS WHO grade 3 GliO co-cultures. Scale bar, 100μm. **c**, Representative confocal microscopy images of GliO alone or cNS + CNS WHO grade 2 or 3 GliO co-cultures. Double-positive $Ki67^{+}$ $HNA^{+}$ proliferating cells are quantified. HNA marks human cells within cNS (mouse) + GliO (human) co-cultures. Top: n=36 GliO regions from two patients and n=23 patient-matched cNS + GliO regions. Bottom: n=41 GliO regions from two patients, n=55 patient-matched cNS + GliO regions. Scale bar, 100μm. **d**, Representative confocal microscopy images showing GliO invasion into cNS from cNS + CNS WHO grade 2 or 3 GliO co-cultures. Negative distance values on the x-axis indicate micrometers invaded into the cNS portion of the co-culture. Top: n=5 co-cultures from two patients. Bottom: n=7 co-cultures from two patients. Scale bar, 100μm. **e**, Representative confocal microscopy images showing $Homer1^{+}$ $Synapsin1^{+}$ synaptic puncta in cNS alone or in cNS + CNS WHO grade 2 or 3 GliO co-cultures. Colocalized of Homer1 and Synapsin1 per $DAPI^{+}$ nuclei is quantified. n=24 cNS regions, n=25 cNS + CNS WHO grade 2 GliO regions, and n=27 cNS + CNS WHO grade 3 GliO regions. Scale bar, 10μm. **f**, MEA analysis of weighted mean firing rate, synchronization index, and average burst frequency in DMSO-treated control (n=11 co-cultures, 4 patients) versus 50μM gabapentin (GBP)-treated cNS + CNS WHO grade 2 and 3 GliO co-cultures (n=13 co-cultures, 4 patients). **g**, MEA analysis of weighted mean firing rate, synchronization index, and average burst frequency in DMSO-treated control (n=5 co-cultures, 3 patients) versus 50μM meclofenamic acid (MFA)-treated cNS + CNS WHO grade 2 and 3 GliO co-cultures (n=6 co-cultures, 3 patients). **h**, Quantification of double-positive $Ki67^{+}$ $HNA^{+}$ proliferating cells after control (n=107 regions from 4 patients, GBP (n=57 regions from 3 patients) or MFA (n=77 regions from 3 patients) treatment of cNS + CNS WHO grade 2 and 3 GliO co-cultures. Lines represent means and error bars represent the standard error of the means. Student's t-tests, *p<0.05, **p<0.01, ***p<0.0001

## Supplementary Files

This is a list of supplementary files associated with this preprint. Click to download.

- MirchiaOtenSupplementaryVideo1v7.avi
- MirchiaOtenSupplementaryVideo2v7.avi
- MirchiaOtenSupplementaryVideo3v7.avi
- MirchiaOtenSupplementaryTablesv7.xlsx
- MirchiaOtenEDFigsv7.docx
